# Alpha and theta audiovisual interventions in a reflective chamber demonstrate acute effects on stress and burnout

**DOI:** 10.1038/s41746-026-02555-z

**Published:** 2026-03-28

**Authors:** Aidan Cone, Sam Zuzick, Tiffany Durinski, Eric Yates, Ninette Simonian, Nicco Reggente

**Affiliations:** Institute for Advanced Consciousness Studies, Santa Monica, CA USA

**Keywords:** Health care, Neuroscience, Psychology, Psychology

## Abstract

Escalating stress prevalence, particularly among essential service personnel whose cognitive compromise threatens public welfare, necessitates rapid, accessible non-pharmacological interventions. Individuals who struggle with endogenous cultivation of beneficial neuropsychological states may benefit from passive exogenous stimulation. This uncontrolled exploratory investigation (*n* = 74) evaluated single-session rhythmic audiovisual stimulation protocols delivered within an immersive reflective chamber (MindGym), wherein employed individuals with self-reported high-stress received randomized assignment to alpha (9–11 Hz) or theta (4–7 Hz) conditions, the sole intervention difference being stimulation frequency of synchronized stroboscopic light and binaural beats during an 11.5-min exposure. Both protocols were well-tolerated and associated with substantial acute improvements in anxiety, mood disturbance, flow states, and vitality, alongside moderate enhancements in perceived stress and purpose in life. No between-protocol differences emerged, though baseline mood disturbance predicted preferential theta-enhancement of existential purpose. Neurophysiological entrainment evidence was inconclusive given consumer-grade instrumentation. These findings establish feasibility warranting controlled trials with active comparisons and follow-up.

## Introduction

Stress prevalence has reached epidemic proportions globally, with well-documented pathological trajectories^1^ and escalating prevalence^2^—evidenced by 85% of countries reporting increased emotional distress between 2008 and 2020^3^ while American stress prevalence escalated from 33% to 49% between 2003 and 2023^4^. The often insidious nature of chronic stress manifests through multifaceted deterioration spanning psychological, cognitive, and physiological domains. Stress-related pathology encompasses heightened suicide risk^5^, substance abuse disorders^6^, and cognitive deterioration, including impaired working memory and attention^7^, response inhibition deficits^8^, and reduced cognitive flexibility^9^. These manifestations reflect underlying neurobiological dysregulation involving HPA-axis hyperactivity and cortisol desensitization^10,11^, culminating in hippocampal neurotoxicity, dendritic retraction, and eventual volume loss that precipitates memory deficits and mood disorders^12–16^.

This mounting psychological burden transcends individual discomfort to encompass systemic societal vulnerabilities, generating cascading risks that threaten civilian welfare through stress-compromised decision-making among essential service personnel operating in life-critical contexts: military personnel experiencing compromised combat readiness and elevated burnout risk^17,18^, healthcare providers including nurses^19^ and physicians^20^, and first responders^21^.

This escalating epidemiological trajectory necessitates targeted therapeutic interventions that can mitigate stress-induced pathophysiology while preserving operational capacity, as stress-compromised decision-making among essential service personnel constitutes both a public health crisis and a strategic national security vulnerability requiring evidence-based solutions that enhance cognitive resilience and operational effectiveness while safeguarding civilian welfare. Contemporary treatment modalities demonstrate variable efficacy across diverse intervention frameworks. Pharmacological approaches encompass both conventional anxiolytics and emerging psychedelic-assisted therapies, the latter demonstrating the capacity to induce altered states of consciousness that fundamentally reconfigure experiential patterns and promote updating of maladaptive beliefs^22^. However, these interventions carry substantial operational constraints: conventional medications risk cognitive impairment and increased risk of dementia^23^ and dependency^24^, while psychedelic interventions often require extended periods of functional incapacitation, present potential for false insights and strengthening of maladaptive beliefs^25^, and systematically exclude individuals with psychotic predispositions, concurrent antidepressant regimens, or cardiovascular complications^26^. Behavioral interventions, particularly mindfulness-based approaches, demonstrate efficacy for burnout prevention^27^ yet reveal a therapeutic paradox: populations experiencing the greatest benefit—those with depressive rumination, cognitive reactivity, or chronic pain^28–30^—simultaneously exhibit the most pronounced adherence difficulties, even among highly motivated practitioners^31,32^. Implementation barriers manifest as substantial dropout rates—19.1% weighted average across 114 studies^33^—particularly problematic for populations experiencing stress-compromised cognitive resources and demanding operational schedules that systematically undermine sustained contemplative practice requirements.

These clinical and practical limitations highlight the imperative for rapid-onset, non-pharmacological interventions that circumvent traditional therapeutic constraints while maintaining operational readiness. Non-invasive, non-pharmacological induction of beneficial neural oscillatory patterns represents a promising alternative approach. Electroencephalography (EEG) studies demonstrate that alpha oscillations (9–11 Hz) reflect relaxed wakefulness and cortical inhibition, while theta oscillations (4–7 Hz) facilitate memory consolidation, internal attention, and meditative states^34,35^. Chronic stress systematically dysregulates these protective neural signatures, manifesting as reduced alpha power and altered theta activity that compromise resting-state networks and cognitive-emotional balance^36,37^. Additionally, restoration of theta and alpha activity through targeted interventions correlates with enhanced psychological well-being, reduced anxiety, and improved emotional regulation^38,39^.

Audiovisual stimulation (AVS), particularly steady-state visual evoked potentials (SSVEPs), presents as a promising route to inducing beneficial brain states that may be more elusive to populations suffering from high stress and a risk of burnout. SSVEPs represent a well-established neurophysiological phenomenon wherein rhythmic stroboscopic stimulation at specific frequencies elicits frequency-matched, phase-locked cortical oscillations across a large swath of regions^40,41^.

Brief stroboscopic visual and binaural auditory stimulation protocols demonstrate comparable acute mood-regulating benefits to traditional meditation practices while requiring approximately 25% of the temporal investment and exhibiting substantially reduced attrition risk^42^, suggesting that rhythmic AVS represents a highly accessible “plug-and-play” modality for rapidly inducing beneficial neural oscillatory patterns and accompanying affective states without the sustained attentional demands that systematically compromise traditional contemplative approaches in high-stress, working adult populations with an elevated risk of burnout.

Such rhythmic AVS effects stand to be substantially enhanced through immersive, multisensory environments that promote enhanced attentional and emotional engagement^43^. MindGym (Lumena, Inc.) exemplifies this technological approach—a reflective chamber featuring LED arrays at cube vertices and mirrored surfaces capable of generating mood-elevating experiences^44^. The potential therapeutic mechanisms underlying immersive AVS may manifest through multiple pathways, including awe induction via novel sensory stimulation, SSVEP-based neural entrainment at targeted frequencies, multimodal sensory integration, or synergistic interactions among these processes—warranting a systematic investigation of relative contributions to outcomes. Furthermore, individual neurophysiological variability necessitates personalized protocols that accommodate neurodiversity, acknowledging that some individuals may demonstrate different optimal therapeutic responsiveness through, for example, alpha vs. theta-based protocols^31^.

This current pilot investigation represents the first examination of rhythmic AVS within an immersive reflective chamber environment (MindGym), with aims of establishing proof-of-concept therapeutic benefit, characterizing neurophysiological correlates, and identifying trait and state moderators of efficacy. Specifically, we sought to generate preliminary effect size estimates for the differential therapeutic capacity of alpha versus theta frequency protocols while investigating evidence of frequency-specific neural entrainment (SSVEPs) in working adults exhibiting elevated stress and burnout vulnerability. This study employed a phenotypic approach designed to identify trait-level moderators that determine optimal intervention responsiveness—specifically focusing on which psychological characteristics predict superior therapeutic outcomes from alpha-frequency versus theta-frequency protocols. Taken together, this methodological framework addresses a fundamental question in neurotechnology applications: whether therapeutic relief can be achieved through one-size-fits-all frequency targeting, or whether individual phenotypic characteristics represent critical determinants of intervention responsiveness.

This preliminary study examined acute affective changes and broader well-being outcomes following rhythmic AVS in working adults exhibiting elevated stress and burnout vulnerability. We assessed intervention-induced changes across validated state measures—including anxiety, mood disturbance, affect, vitality, and flow—alongside trait-level indices of perceived stress^45^ and purpose in life (PIL)^46^. This dual assessment framework enabled examination of both immediate felt experiences and downstream well-being constructs theorized to reflect cumulative effects of sustained stress exposure in occupational populations.

Perceived stress scale (PSS) and PIL represent inversely correlated constructs^47^ that together characterize burnout vulnerability more comprehensively than either alone. PIL—defined as “having a sense of meaning and purpose regarding one’s activities as well as an overall sense that life is meaningful”^48^—buffers against burnout-related outcomes across occupational populations, reducing emotional exhaustion in firefighters^49^ and burnout in physicians^50^, while predicting superior emotional recovery from negative stimuli^51^, suggesting complementary resilience mechanisms beyond mere stress reactivity. Given the current study’s high-stress, employed cohort, we assessed both stress reduction (symptom amelioration) and PSS/PIL changes (resilience augmentation). To ground these primary outcomes within the broader burnout construct space, we examined baseline correlations with validated burnout inventories collected during screening, providing a foundation for interpreting intervention effects in relation to burnout trajectories.

## Results

### Psychological measures

Results are presented in order of evidentiary strength, beginning with acute state effects, followed by the hypothesized downstream outcome measures (PSS, PIL), which should be interpreted with appropriate caution given their broader scope and susceptibility to confounding.

The screening measures revealed a sample experiencing substantial burnout-related distress (Table 1). PIL scores (*M* = 66.43, SD = 13.12) indicated lowered life purpose scores, while PSS scores (*M* = 22.74, SD = 5.62) indicated moderate-to-high stress levels consistent with our eligibility threshold (PSS ≥ 14). Burnout inventory scores across multiple validated instruments (BBI, MBI, CBI, OBI) demonstrated mostly elevated exhaustion, cynicism, and feelings of inadequacy.

**Table 1:** Descriptive statistics for stress and burnout related screening measures (*N* = 69)

Measure	M	SD	Min	Max	Range
Purpose in life (PIL)	66.43	13.12	41.00	94.00	53.00
Perceived stress scale (PSS)	22.74	5.62	14.00	38.00	24.00
BBI exhaustion	11.01	3.58	3.00	18.00	15.00
BBI cynicism	11.48	4.02	3.00	18.00	15.00
BBI inadequacy	11.80	3.45	5.00	18.00	13.00
MBI exhaustion	20.30	8.03	0.00	30.00	30.00
MBI cynicism	18.75	8.12	0.00	30.00	30.00
MBI professional efficacy	23.68	5.86	11.00	35.00	24.00
Copenhagen Burnout Inventory	57.99	19.39	15.28	100.00	84.72
Oldenburg Burnout Inventory	39.91	7.23	27.00	57.00	30.00

*BBI* Bergen Burnout Indicator, *MBI* Maslach Burnout Inventory, *PIL* purpose in life, *PSS* perceived stress scale.

Strong correlations between burnout measures and our outcome measures validated PSS and PIL as burnout-relevant proxies (Table 2). As expected, all burnout indicators showed negative correlations with PIL (*r* = −0.44 to −0.58, all *p* < 0.001) and positive correlations with Perceived Stress (*r* = 0.38 to 0.63, all *p* < 0.01), with the exception of MBI Professional Efficacy, which demonstrated the predicted inverse pattern (positive correlation with PIL: *r* = 0.41, *p* < 0.001; negative correlation with PSS: *r* = −0.36, *p* < 0.01). PIL and PSS themselves showed a strong negative correlation (*r* = −0.55, 95% CI [−0.69, −0.35], *p* < 0.001), confirming their conceptual relationship as opposing indicators of psychological well-being and distress.

**Table 2:** Baseline correlations between outcomes and burnout measures (*N* = 69)

Burnout measure	*r* (PIL)	95% CI	*r* (PSS)	95% CI
BBI exhaustion	−0.55***	[−0.70, −0.37]	0.51***	[0.31, 0.67]
BBI cynicism	−0.55***	[−0.69, −0.35]	0.52***	[0.32, 0.67]
BBI inadequacy	−0.44***	[−0.61, −0.22]	0.38**	[0.16, 0.57]
MBI exhaustion	−0.52***	[−0.67, −0.33]	0.55***	[0.35, 0.69]
MBI cynicism	−0.46***	[−0.63, −0.26]	0.52***	[0.32, 0.67]
MBI professional efficacy	0.41***	[0.20, 0.59]	−0.36**	[−0.55, −0.13]
Copenhagen Burnout Inventory	−0.58***	[−0.72, −0.40]	0.57***	[0.38, 0.71]
Oldenburg Burnout Inventory	−0.58***	[−0.72, −0.40]	0.63***	[0.46, 0.75]

*PIL* purpose in life, *PSS* perceived stress scale, *BBI* Bergen Burnout Indicator, *MBI* Maslach Burnout Inventory. PIL-PSS correlation: *r* = −0.55, 95% CI [−0.69, −0.35], *p* < 0.001.

^**^p < 0.01, ^***^p < 0.001.

To further understand the potential for regression to the mean in our outcome measures, we examined the correlation between baseline PSS scores and PSS change scores (post minus pre), revealing a marginally significant negative correlation (*r* = −0.24, *p* = 0.047). This pattern suggests that participants with higher baseline stress showed numerically greater stress reduction. This relationship admits multiple interpretations: genuine ceiling/floor effects wherein those with greater initial distress have more room for improvement, regression to the mean as a statistical artifact, or differential intervention responsiveness as a function of baseline severity. Without appropriate control conditions, distinguishing among these mechanisms remains impossible, underscoring the preliminary nature of these findings and the necessity of controlled replication.

Nearly all state measures demonstrated significant main effects of time (Table 3), indicating that both alpha and theta conditions were associated with substantial improvements from pre- to post-intervention, with no significant time × condition interactions or between-group differences emerging, thus suggesting equivalent changes observed across stimulation protocols.

**Table 3:** Results of repeated measures ANOVA in state measures

Measure category	Specific measure	*F*-statistic (1, 67)	*p*-value	Effect size (*η²*_*G*_*;* Cohen’s *d*)	Bonferroni corrected *p*-value
Anxiety	STAI-state	95.862	<0.001	0.324; 1.365 [95% CI, 1.001–1.730]	<0.001
	Anxiety emoji scale	20.252	<0.001	0.115; 0.713 [95% CI, 0.374–1.052]	<0.001
Depression & mood	POMS depression	74.687	<0.001	0.258; 1.162 [95% CI, 0.827–1.497]	<0.001
	POMS total mood disturbance	58.491	<0.001	0.261; 1.172 [95% CI, 0.811–1.534]	<0.001
	POMS tension	53.559	<0.001	0.219; 1.045 [95% CI, 0.708–1.382]	<0.001
	POMS fatigue	45.771	<0.001	0.185; 0.941 [95% CI, 0.619–1.262]	<0.001
	POMS confusion	36.949	<0.001	0.157; 0.850 [95% CI, 0.535–1.166]	<0.001
	POMS anger	34.785	<0.001	0.193; 0.963 [95% CI, 0.598–1.329]	<0.001
	POMS vigor	1.087	0.301	0.005; −0.098 [95% CI, −0.393–0.124]	N/A
	PANAS negative affect	33.735	<0.001	0.034; 0.848 [95% CI, 0.522–1.174]	<0.001
Flow states	FSS total	54.016	<0.001	0.151; −0.832 [95% CI, −1.100 to −0.564]	<0.001
	FSS fluency of performance	46.893	<0.001	0.155; −0.844 [95% CI, −1.130 to −0.558]	<0.001
	FSS absorption by activity	29.071	<0.001	0.084; −0.598 [95% CI, −0.842 to −0.354]	<0.001
Positive affect	PANAS positive affect	9.505	0.003	0.034; −0.369 [95% CI, −0.616 to −0.122]	0.045
	Subjective vitality scale	17.498	<0.001	0.059; −0.493 [95% CI, −0.743 to −0.243]	<0.001

**Fig. 1:**
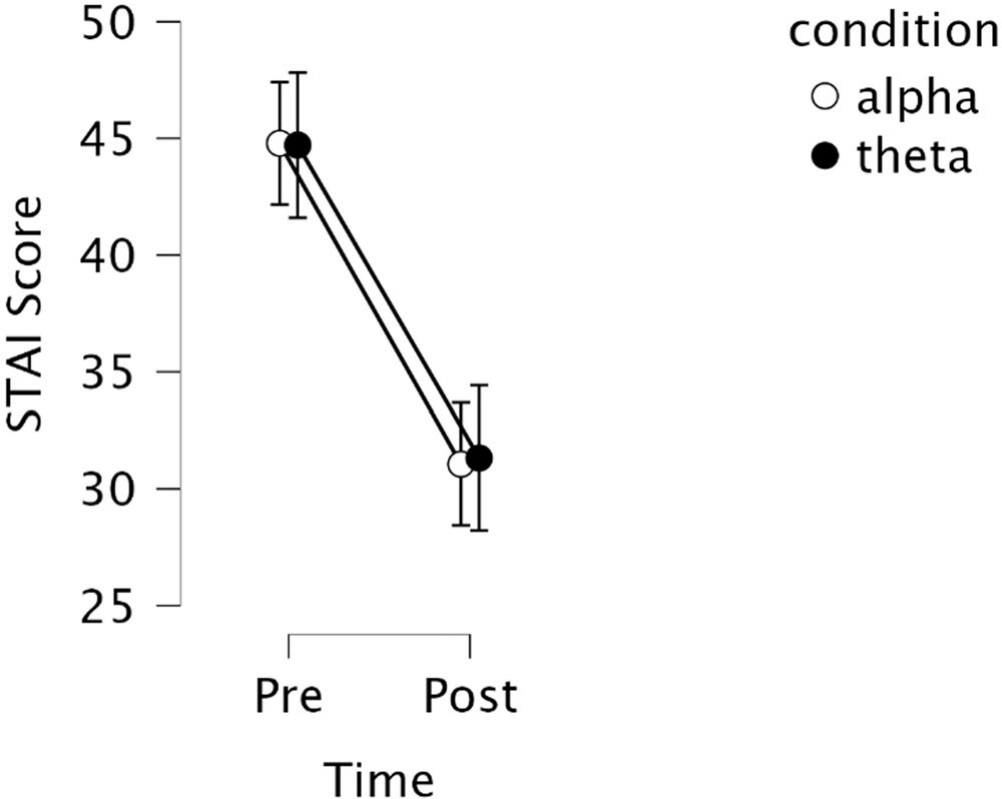
Mean state anxiety scores from the state-trait anxiety inventory (STAI) for alpha and theta conditions at pre- and post-intervention time points. Both conditions showed substantial reductions in state anxiety from pre- to post-intervention, with no significant differences between conditions. Lower scores represent less reported anxiety. Error bars represent standard error of the mean.

Both anxiety measures demonstrated robust pre-to-post intervention improvements: STAI-State (Fig. 1; *F*(1,67) = 95.862, *p*_*bonf*_ < 0.001, *η²*_*G*_ = 0.324) and the Anxiety Emoji Scale (*F*(1,67) = 20.252, *p*_*bonf*_ < 0.001, *η²*_*G*_ = 0.115). The particularly large effect size for STAI-State anxiety reduction observed following the intervention represents one of the most pronounced effects observed across all measured domains, emphasizing the intervention’s strong association with anxiolytic effects.

**Fig. 2:**
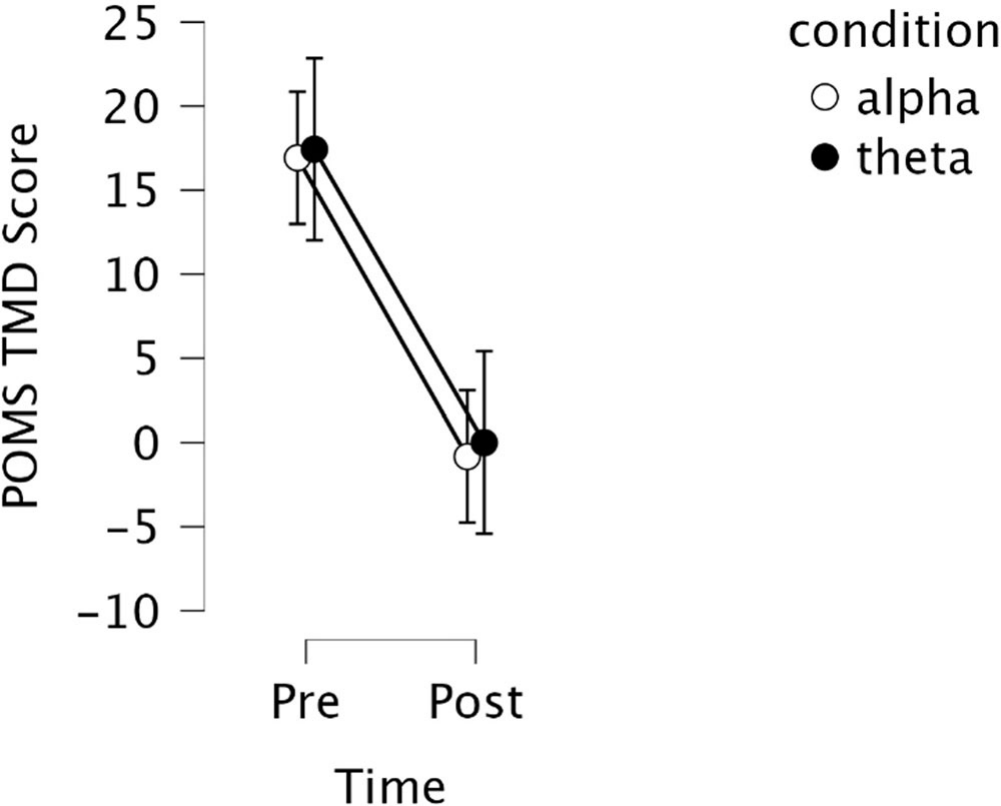
Mean profile of mood disturbance (POMS) total mood disturbance (TMD) scores before and after 235-minute audiovisual stimulation for alpha and theta conditions. Both conditions demonstrated substantial TMD reductions from approximately 17 to near zero. Lower scores indicate improved mood states. Error bars represent standard error of the mean.

All mood measures also showed significant improvements: Profile of Mood States (POMS) Depression (*F*(1,67) = 74.687, *p*_*bonf*_ < 0.001, *η²*_*G*_ = 0.258), POMS Tension (*F*(1,67) = 53.559, *p*_*bonf*_ < 0.001, *η²*_*G*_ = 0.219), POMS Anger (*F*(1,67) = 34.785, *p*_*bonf*_ < 0.001, *η²*_*G*_ = 0.193), POMS Fatigue (*F*(1,67) = 45.771, *p*_*bonf*_ < 0.001, *η²*_*G*_ = 0.185), POMS Confusion (*F*(1,67) = 36.949, *p*_*bonf*_ < 0.001, *η²*_*G*_ = 0.157), POMS Total Mood Disturbance (TMD; Fig. 2; *F*(1,67) = 58.491, *p*_*bonf*_ < 0.001, *η²*_*G*_ = 0.261), PANAS Positive Affect (*F*(1,67) = 9.505, *p*_*bonf*_ = 0.045, *η²*_*G*_ = 0.034), and PANAS Negative Affect (*F*(1,67) = 33.735, *p*_*bonf*_ < 0.001, *η²*_*G*_ = 0.034).

Flow measures also demonstrated significant increases: Flow State Scale (FSS) Absorption by Activity (*F*(1,67) = 29.071, *p*_*bonf*_ < 0.001, *η²*_*G*_ = 0.084), FSS Fluency of Performance (*F*(1,67) = 46.893, *p* < 0.001, *η²*_*G*_ = 0.155), and FSS Total (*F*(1,67) = 54.016, *p*_*bonf*_ < 0.001, *η²*_*G*_ = 0.151). Similarly, the Subjective Vitality Scale (SVS) showed a significant increase (*F*(1,67) = 17.498, *p*_*bonf*_ < 0.001, *η²*_*G*_ = 0.059).

The effect sizes indicate that the time effects were particularly strong for POMS Depression (*η²*_*G*_ = 0.258, Cohen’s *d* = 1.162 [95% CI, 0.827–1.497]), POMS TMD (*η²*_*G*_ = 0.261, Cohen’s *d* = 1.172 [95% CI, 0.811–1.534]), and FSS Total (*η²*_*G*_ = 0.151, Cohen’s *d* = −0.832 [95% CI, −1.100 to −0.564]), demonstrating these measures showed large effect size changes from pre to post across both conditions. All time effects remained significant after applying Bonferroni correction for multiple comparisons across all 15 state comparisons (all *p*_*bonf*_ < 0.004), indicating robust improvements across anxiety, mood, flow, and vitality domains.

Repeated measures Analysis of Variance (ANOVA) revealed significant main effects of time for the outcome measures after correction for multiple comparisons (Table 4a), with post hoc analyzes conducted for significant findings. Perceived stress (PSS) decreased significantly from pre- to post-intervention (*F*(1,67) = 28.458, *p* < 0.001, *p*_*bonf*_ < 0.001, *η²*_*G*_ = 0.051, *d* = 0.458 [95% CI, 0.270, 0.647]) and PIL increased significantly (*F*(1,67) = 14.496, *p* < 0.001, *p*_*bonf*_ < 0.001, *η²*_*G*_ = 0.071, *d* = −0.547 [95% CI, −0.849–0.245]). No significant main effects of condition were observed for either PSS (*F*(1,67) = 0.073, *p* = 0.788, *p*_*bonf*_ = 0.788, *η²*_*G*_ = < 0.001) or PIL (*F*(1,67) = 0.263, *p* = 0.610, *p*_*bonf*_ = 0.732, *η²*_*G*_ = 0.003). The time × condition interaction for PSS (*F*(1,67) = 3.814, *p* = 0.055, *p*_*bonf*_ = 0.110, *η²*_*G*_ = 0.007) and PIL (*F*(1,67) = 1.474, *p* = 0.229, *p*_*bonf*_ = 0.344, *η²*_*G*_ = 0.008) were also non-significant.

**Table 4:** Results of repeated measures ANOVA in outcome measures

Measure category	Specific measure	*F*-statistic	*p*-value	Effect size (*η²*_*G*_*;* Cohen’s *d*)	Bonferroni corrected *p*-value
Stress	PSS time effect	28.458	< 0.001	0.051; 0.458 [95% CI, 0.270 – 0.647]	<0.001
	PSS condition effect	0.073	0.788	< 0.001; 0.061 [95% CI, −0.380 to 0.510]	0.788
	PSS time × condition	3.814	0.055	0.007	0.110
Purpose	PIL time effect	14.496	<0.001	0.071; −0.547 [95% CI, −0.849 to −0.245]	<0.001
	PIL condition effect	0.263	0.610	0.003; 0.099 [95% CI, −0.287 to 0.486]	0.732
	PIL time × condition	1.474	0.229	0.008	0.344

Time effects pertain to the repeated measures from questionnaire responses during screening and then again following the intervention. Condition effects pertain to alpha vs. theta group comparisons. Post-intervention phenomenological measures revealed no significant between-group differences (Table 5). The Emotional Breakthrough Inventory showed no significant difference between alpha and theta conditions (*t*(67) = −1.075, *p* = 0.286, *d* = −0.259), nor did the Toronto Mindfulness Scale (*t*(67) = −0.750, *p* = 0.456, *d* = −0.181), indicating equivalent phenomenological profiles across frequency-specific protocols.

Post hoc analyzes revealed significant improvements, collapsing across both conditions, with reductions in PSS (Alpha: −3.72; Theta: −1.73) and increases in PIL (Alpha: +4.69; Theta: +9.09). Independent samples *t*-tests on change scores (post minus pre) showed a marginally significant difference between groups for reduction in PSS scores (*t*(67) = −1.953, *p* = 0.055, *d* = −0.471), with Alpha participants showing numerically greater stress reduction, but no significant difference for PIL changes (*t*(67) = −1.214, *p* = 0.229, *d* = −0.293).

### Mediation

Mediation analyzes revealed no significant indirect effects of condition (alpha vs. theta) through any psychological mediating variables to either outcome (PIL or PSS). The indirect effects of condition on both outcomes were consistently non-significant across all models, indicating that the equivalent changes observed for both interventions were not explained through the measured psychological variables. While several psychological variables showed significant direct relationships with outcomes—such as State-Trait Anxiety Inventory (STAI), FSS and POMS TMD improvements being associated with enhanced PIL (ΔSTAI → ΔPIL: *b* = −0.676, *p* < 0.001; ΔFSS Total → ΔPIL: *b* = 1.384, *p* < 0.001; ΔPOMS TMD → ΔPIL: *b* = −0.280, *p* = 0.031), and reductions in PANAS Negative Affect correlating with reductions in PSS scores (ΔPANAS NA → ΔPSS: *b* = −0.184, *p* = 0.009) —these relationships were independent of intervention condition. These correlations indicate that psychological changes co-occur with improvements in primary outcomes, though the similar pattern across conditions suggests both interventions may be associated with comparable psychological change processes. The absence of mediation pathways suggests that if differential effects between protocols exist, they may operate through mechanisms not fully captured by these self-report psychological measures, or may reflect measurement limitations inherent in the current study design.

**Table 5:** Results of between group *t*-tests in post-intervention outcome measures

Measure	*t*-statistic	*p*-value	Cohen’s *d*
Emotional breakthrough inventory	−1.075	0.286	−0.259
Toronto mindfulness scale	−0.750	0.456	−0.181

*T*-tests of the Emotional Breakthrough Inventory (EBI) and Toronto Mindfulness Scale (TMS). Results show no statistical significance between the two conditions in either measure.

### Moderation

We examined whether pre-intervention psychological states moderated the effects of alpha versus theta on outcome measures. Several significant findings emerged as interaction effects between condition and baseline measures, indicating differential moderating patterns between the two intervention types (Table 6).

**Table 6:** State predictors moderation of change in outcome measure

Outcome	Significant moderators	Key finding
Purpose in life (PIL)	POMS TMD (*p*_*FDR*_ = 0.033) POMS anger (*p*_*FDR*_ = 0.019) POMS Depression (*p*_*FDR*_ = 0.033) POMS confusion (*p*_*FDR*_ < 0.001)	Several pre-intervention POMS subscales and total scale significantly moderate changes in PIL when interacting with condition
Perceived stress scale (PSS)	Non-significant	No significant findings

**Fig. 3:**
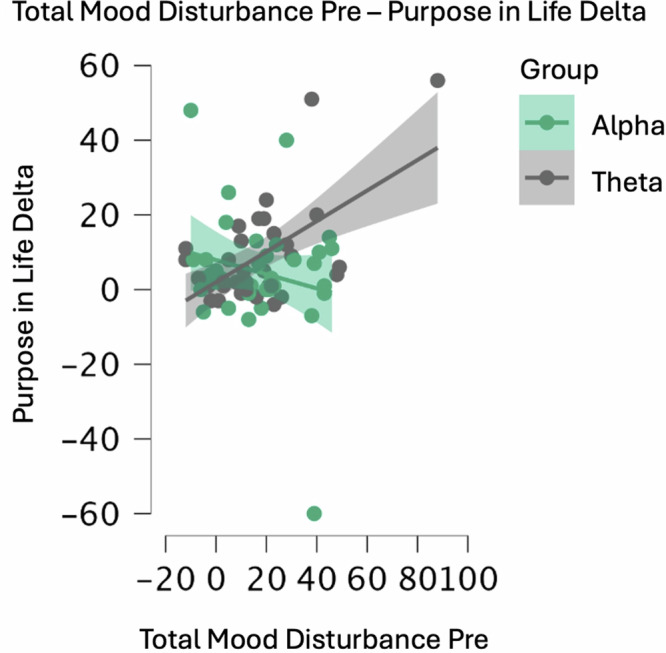
Moderation of purpose in life outcomes by pre-intervention mood disturbance across intervention conditions. Scatterplot shows the relationship between baseline Profile of Mood States Total Mood Disturbance scores (*x*-axis) and change in Purpose in Life scores from pre- to post-intervention (*y*-axis) for alpha (green) and theta (gray) conditions. Lines represent fitted regression slopes with 95% confidence intervals (shaded areas). A significant interaction effect was observed (*p*_FDR_ = 0.033), indicating that pre-intervention mood disturbance differentially predicted Purpose in Life improvements between conditions. Purpose in Life Delta = post-intervention Purpose in Life score minus pre-intervention Purpose in Life score; positive values indicate improvement in sense of purpose.

Significant findings included moderation of the relationship between condition and PIL Outcomes by POMS TMD (Fig. 3), Anger, Depression, and Confusion. Participants with higher baseline mood disturbance showed greater PIL improvements following the theta condition compared to the alpha condition, while those with lower baseline mood disturbance showed similar improvements following both interventions.

Secondary exploratory analyzes using a median split on baseline POMS TMD examined whether intervention effects differed by initial distress level (See Supplementary Materials: Supplementary Tables 1 and 2). A 2 × 2 ANOVA analysis revealed significant conditional interaction between POMS TMD and PIL Delta (*p* = 0.046) and FSS Total Delta approached significance (*p* = 0.07). Post-hoc independent sample *t*-tests between High and Low POMS TMD groups revealed theta showed greater improvement in PIL in individuals with higher baseline POMS TMD (*p*_*bonf*_ = 0.023, Cohen’s *d* = −0.815). Additionally, individuals with High baseline POMS TMD also showed greater improvement in FSS Total Delta with the theta condition (*p*_*bonf*_ = 0.021, Cohen’s *d* = −0.824). Interestingly, a continuous moderation analysis further showed no significant interaction between POMS TMD group and intervention condition for PIL change scores (*p* = 0.140) and FSS Total delta scores (*p* = 0.110).

Analysis of PSS outcomes identified several pre-intervention predictors including Flow State Scale Total scores, FSS Fluency of Performance subscale ratings, anxiety emoji assessments, and PANAS Negative Affect levels; however, none remained significant following False Discovery Rate (FDR) correction. Alpha protocol participants demonstrated greater mean stress reduction (*M* = −3.72, SD = 3.10) compared to theta participants (*M* = −1.73, SD = 5.20). No baseline state measures significantly moderated Emotional Breakthrough Inventory (EBI) or Toronto Mindfulness Scale (TMS) outcomes following either condition.

No trait variables collected during the screening significantly moderated outcome variables.

### Neurophysiological results

To examine changes over time in heart rate (HR) and heart rate variability (HRV), we employed repeated measures ANOVA which resulted in no significant changes over time comparing the pre-rest and post-rest stages (See Supplementary Materials: Supplementary Table 3). To examine EEG changes over time, we employed repeated measures ANOVA which resulted in a significant difference in Alpha power comparing the pre-rest and post-rest stages, but did not survive Bonferroni correction (See Supplementary Materials: Supplementary Tables 4 and 5).

All independent sample *t*-tests for analysis of physiological measures (HR and HRV) revealed no statistical differences between the Alpha and Theta groups after correcting for multiple comparisons (See Supplementary Materials: Supplementary Table 6). All independent sample *t*-tests for analysis of EEG measures (including power band ratios) revealed no statistical differences between the Alpha and Theta groups after correcting for multiple comparisons (See Supplementary Materials: Supplementary Table 6). Mediation analyzes were conducted to examine whether changes in EEG band powers served as mediating mechanisms linking treatment condition to psychological measure outcomes. However, no significant indirect effects were observed across any of the EEG frequency bands examined, indicating that the measured neural oscillatory changes did not significantly mediate the relationship between treatment condition and psychological measure improvements. These findings suggest that the psychological changes observed following both conditions may reflect alternative neurobiological pathways not captured by the specific EEG frequency bands analyzed, or that the associations occur through more complex neural network interactions beyond simple power changes in isolated frequency ranges.

Several potential moderation effects of baseline EEG measures on treatment outcomes were observed at the uncorrected statistical level; however, none of these effects survived correction for multiple comparisons using the FDR method (all *p*_*FDR*_ > 0.05). Additionally, baseline alpha/theta ratios showed a significant negative correlation with intervention-related changes of the same (*r* = −0.38, *p* = 0.002), suggesting a regression-to-the-mean effect where the intervention may be associated with normalization rather than uniformly alter neurophysiological activity (See Supplementary Materials: Supplementary Fig. 1).

## Discussion

This pilot investigation examined whether AVS—binaural beats synchronized with stroboscopic light at alpha (9–11 Hz) vs. theta (4–7 Hz) frequencies—delivered through an immersive reflective chamber (MindGym) could meaningfully impact stress, burnout mitigation, and psychological well-being in 74 employed participants experiencing burnout risk. Both 11.5-min protocols, alternating between traditional closed-eye periods and open-eye segments within the chamber’s mirrored environment, demonstrated preliminary evidence supporting feasibility and tolerability: zero adverse events were reported, zero participants terminated early, and 100% of enrolled participants completed the protocol. However, a formal assessment of tolerability using systematic adverse event monitoring instruments was not conducted. While no significant differences in acute outcomes emerged as a function of intervention protocol (alpha vs. theta AVS), the magnitude of pre-to-post changes across burnout-relevant affective domains was remarkable, though warranting caution given the lack of a control group. Anxiety demonstrated the most substantial reductions, with the STAI-State exhibiting large effects (*p* < 0.001, *d* = 1.37) alongside significant improvements on the Anxiety Emoji Scale (*p* < 0.001, *d* = 0.71).

Mood disturbance showed comparable amelioration across the POMS, with large effects for Depression (*p* < 0.001, *d* = 1.16), TMD (*p* < 0.001, *d* = 1.17), Tension (*p* < 0.001, *d* = 1.05), and substantial effects for Fatigue (*p* < 0.001, *d* = 0.94), Anger (*p* < 0.001, *d* = 0.96), and Confusion (*p* < 0.001, *d* = 0.85). The PANAS Negative Affect subscale similarly demonstrated large reductions (*p* < 0.001, *d* = 0.85). Notably, POMS Vigor remained unchanged (*p* = 0.301), suggesting specific, non-sedative anxiolytic and mood-stabilizing effects rather than generalized arousal modulation.

Flow states increased substantially across Flow State Scale metrics, with moderate-to-large effects for Total Flow (*p* < 0.001, *d* = 0.83), Fluency of Performance (*p* < 0.001, *d* = 0.84), and Absorption by Activity (*p* < 0.001, *d* = 0.60). Positive affect demonstrated modest but significant enhancement on both the PANAS Positive Affect subscale (*p* = 0.003, *d* = 0.37) and SVS (*p* < 0.001, *d* = 0.49). The vitality enhancement merits particular attention, given evidence that vitality mediates the relationship between self-efficacy and burnout resistance^52^, with higher vitality predicting sustained work engagement even under stressful conditions. This finding gains additional significance considering burnout may represent a psychophysiologically “sticky” state that rarely improves without substantial environmental change, such as job departure^53^. Demonstrating initial measurable improvement in this high-stress population suggests that even brief, single-session interventions may substantially “move the needle” on traditionally resistant burnout indicators—a particularly salient finding for populations wherein any measurable relief from chronic stress represents meaningful therapeutic progress. Collectively, these patterns suggest associations between brief AVS exposure and comprehensive acute psychological benefit spanning both distress reduction and eudaimonic enhancement.

Beyond these acute state changes, outcome measures collected at screening and reassessed post-intervention—PIL and PSS—demonstrated significant pre-to-post improvements. PSS was associated with moderate reductions (*p* < 0.001, *d* = 0.46), while PIL showed moderate increases (*p* < 0.001, *d* = 0.55). These measures were selected to jointly characterize burnout vulnerability: PSS captures felt affective experience of stress burden, while PIL—defined as “having a sense of meaning and purpose regarding one’s activities as well as an overall sense that life is meaningful”^48^—indexes resilience-conferring meaningfulness that buffers against burnout across occupational populations^49,50^. As inversely correlated constructs^47^, PSS and PIL together provide a more comprehensive assessment of burnout vulnerability than either alone.

The interpretation of PSS and PIL changes as burnout-relevant receives empirical support from their strong baseline correlations with comprehensive burnout inventories collected during screening. PSS demonstrated substantial positive associations with all burnout indicators (*r* = 0.38 to 0.63, all *p* < 0.01), while PIL showed robust negative associations (*r* = −0.44 to −0.58, all *p* < 0.001), with shared variance ranging from 14% to 40%. This substantial overlap—particularly the ~26% average shared variance—provides an empirical foundation for cautiously inferring that observed PSS and PIL improvements may reflect meaningful shifts in underlying burnout-related psychological functioning, though direct assessment of burnout inventories at post-intervention would be required to establish this correspondence definitively (see Future Directions).

The observed effect sizes in this pilot study appeared comparable to some established non-pharmacological interventions, though direct comparisons should be interpreted cautiously given the absence of a control group in the present study. The PSS reduction (*d* = 0.458) was numerically similar to or larger than brief mindfulness interventions (*d* = 0.37)^54^, while anxiety improvements were larger than those reported from 8-week meditation programs (*d* = 0.38)^39^. Additionally, participants showed increases in flow state measures—a phenomenon that has proven difficult to reliably induce through experimental manipulation^55^, despite flow’s recognized importance for performance and well-being. However, these comparisons are limited by key methodological differences: the cited interventions employed randomized controlled designs with active or passive control conditions, whereas the present study utilized a pre-post design without a control group. The observed changes may therefore reflect factors beyond the specific intervention components (e.g., expectancy effects, natural mood fluctuation, regression to the mean).

Surprisingly, we found no evidence supporting neural entrainment as the primary therapeutic mechanism. Neither protocol was associated with significant changes in target frequency band power from pre- to post-intervention, nor did post-intervention EEG profiles differ between groups despite frequency-specific stimulation (all *p*_*FDR*_ > 0.05). However, this null finding likely reflects measurement sensitivity limitations rather than the absence of the phenomenon itself. Extensive literature demonstrates that rhythmic visual stimulation reliably induces frequency-specific neural entrainment when measured with research-grade equipment featuring occipital electrode coverage and adequate spatial sampling^40,41,56^. Compounding the sensitivity concerns of consumer-grade equipment, the Muse-S’s frontal-temporal electrode montage (AF7, AF8, TP9, TP10) provides no coverage of occipital and parieto-occipital regions—precisely the locations where SSVEPs demonstrate maximal amplitude^41,56^. Given this instrumentation constraint, neural entrainment remains a plausible therapeutic mechanism that our equipment lacked the sensitivity to detect. Critically, the absence of detectable entrainment signatures does not diminish the robust psychological improvements observed across both conditions; rather, it leaves the underlying neurobiological mechanism empirically unresolved and invites consideration of alternative neurophenomenological pathways.

For example, the immersive reflective environment itself may constitute a primary therapeutic mechanism through awe induction. The MindGym chamber elicits substantial awe (*M* = 109.85 on the AWE-S)^44^, numerically exceeding VR environments specifically designed to evoke awe and goosebumps (*M* = 79.7)^57^. This awe response engages pathways robustly associated with enhanced meaning-making^58^, reduced sympathetic activation^59^, and improved well-being^60^—mechanisms proposed to underlie therapeutic effects in psychedelic-assisted psychotherapy through self-transcendence and “small self” phenomenology^61^. Alternatively, synchronized AVS may trigger relaxation via subcortical pathways^62^ or network-level reorganization not captured by our limited spectral analysis. The therapeutic equivalence observed across stimulation frequencies reinforces the possibility that immersive multisensory context, coupled with rhythmic AVS, rather than frequency-specific entrainment, constitutes a primary active ingredient. However, the STAI-State reductions in the current study (13.01 points) substantially exceeded those in MindGym-only conditions (3.89 points)^44^—a 3.3-fold numerical enhancement suggesting that rhythmic AVS protocols may provide additive or multiplicative therapeutic benefit beyond environmental immersion alone.

Despite mechanistic uncertainty, exploratory analyzes revealed some potential frequency-dependent dissociations that suggest distinct neurotherapeutic profiles. Outcome analyzes revealed robust improvements across both protocols, yet post-hoc examination unveiled a nuanced dissociation: alpha stimulation was associated with numerically superior stress reduction (ΔPSS = −3.72 vs. −1.73, *p* = 0.055), while theta stimulation was associated with a more pronounced enhancement of existential purpose (ΔPIL = +9.09 vs. +4.69). The pattern extended to state measures, where theta consistently yielded numerically superior improvements across POMS Depression, Tension, Anger, and Fatigue subscales, as well as flow state enhancement (+6.091 vs +4.361). While these differences failed to achieve conventional significance thresholds and should be considered exploratory hypothesis-generating findings, such incremental gains could hold substantial clinical relevance for individuals experiencing burnout and anxiety, where even marginal improvements in mood regulation or positive affect can meaningfully impact functional capacity and quality of life.

This preliminary divergent pattern extended to phenomenological outcomes, with theta participants demonstrating enhanced emotional breakthrough (28.3 vs. 24.3) and systematically, albeit minimal, higher average scores in the TMS: heightened curiosity (15.5 vs. 15.3), decentering (17.2 vs. 15.5), and overall mindful awareness (32.7 vs. 30.8). These potential theta-specific enhancements align with established associations between frontal midline theta and meditative states, whereas alpha-meditation relationships exhibit greater individual variability^63,64^. The observed changes following the interventions, with equivalence between alpha and theta protocols, could reflect response distributions wherein strong and weak responders populate each condition in approximately equal proportions. Such a potential motivates a reconceptualization of population-level, “one-size-fits-all” interventions toward personalized paradigms—replacing random assignment with algorithmic stratification based on individual phenotypic markers encompassing both who participants fundamentally are (trait characteristics) and how they present at intervention onset (state variables). To elucidate potential biomarkers for such personalized protocol selection, we conducted exploratory moderation analyzes examining trait and state (including EEG measures) moderators of therapeutic response.

Despite theoretical expectations that dispositional characteristics—particularly trait mindfulness, openness to experience, or beliefs around the role of personal agency in health outcomes—might moderate receptivity to passive AVS, no trait variables demonstrated significant moderating effects. Similarly, pre-intervention neurophysiological profiles (EEG spectral power across frequency bands) failed to predict differential outcomes between protocols after correcting for multiple comparisons. However, state-dependent psychological variables revealed striking protocol-specific moderation patterns exclusively for existential outcomes. The relationship between intervention condition and PIL enhancement was significantly moderated by multiple baseline mood disturbance indicators—POMS TMD (*p*_*FDR*_ = 0.033), Anger (*p*_*FDR*_ = 0.019), Depression (*p*_*FDR*_ = 0.033), and Confusion (*p*_*FDR*_ < 0.001). Participants presenting with elevated baseline psychological dysfunction showed preferential PIL enhancement associated with theta stimulation, while those with lower baseline disturbance achieved comparable improvements across both protocols. Secondary analyzes further revealed that baseline POMS TMD influenced the relationship between intervention type and PIL outcomes (*p*_*bonf*_ = 0.023, Cohen’s *d* = −0.815) and FSS Total Score (*p*_*bonf*_ = 0.021, Cohen’s *d* = −0.824), with theta-frequency stimulation showing stronger associations with improvements following the intervention among participants with higher baseline distress. This suggests that theta’s apparent advantages for psychological outcomes may be particularly relevant for individuals scoring higher on the POMS TMD scale and warrants further controlled investigation to rule out confounding factors.

This state-dependent, frequency-specific moderation pattern challenges purely non-specific environmental explanations discussed above. If the immersive reflective environment alone drove therapeutic benefits, baseline psychological state should modulate overall response magnitude rather than selectively determining which stimulation frequency proves optimal. The observed frequency-specific interaction—wherein baseline mood disturbance predicts differential responsiveness to theta versus alpha protocols—suggests these interventions may engage distinct psychological or neurobiological pathways that interface differentially with participants’ affective states, even if our instrumentation could not detect underlying neural mechanisms. This pattern is inconsistent with demand characteristics, which would be expected to operate uniformly regardless of protocol assignment or baseline distress levels. While these findings provide preliminary evidence against exclusively non-specific explanations, controlled replication remains necessary to definitively establish intervention-specific effects.

These current preliminary and findings, despite the absence of a control group, hold particular relevance for operational contexts (e.g., military aviators managing G-forces and split-second decisions, special operations personnel sustaining hypervigilance in denied environments, first responders navigating cumulative trauma exposure) where traditional pharmacological stress interventions may induce states of compromised sobriety that prove incompatible with mission demands. Unlike meditation, requiring sustained, disciplined practice or psychotherapy spanning months, AVS may offer immediate stress relief without extensive training pending controlled replication. The technology’s “plug-and-play” nature directly addresses Brandmeyer and Delorme’s observation that Western populations struggle to maintain contemplative practices due to factors “ranging from lack of time to general laziness.”^63^

For military personnel experiencing allostatic load from prolonged deployment cycles, cultural barriers to help-seeking, and limited recovery opportunities, a non-sedative 11.5-min intervention associated with anxiety-reducing and mood-boosting effects comparable to weeks of traditional treatment represents a transformative possibility. The absence of stigma associated with technology-based interventions may facilitate adoption where traditional mental health services encounter resistance rooted in military culture’s emphasis on stoicism and self-reliance.

While promising as in initial proof-of-concept with significant results, this study has limitations warranting interpretative caution and encouraging future work. The absence of control conditions represents a fundamental constraint on causal inference. Without comparator groups (e.g., waitlist, sham stimulation, or random frequency protocols), observed improvements cannot be definitively attributed to the AVS protocols themselves rather than to placebo effects, expectancy, demand characteristics, environmental novelty, or simple rest. This interpretive limitation extends to all outcome measures, as participants’ self-reports may have been influenced by conscious or unconscious adjustment to align with perceived experimental expectations. While the temporal stability of burnout states provides some reassurance against spontaneous fluctuation accounting for observed improvements, this possibility cannot be definitively ruled out without appropriate control conditions. The substantial effect sizes observed, while potentially reflecting genuine intervention effects, may also be inflated by confounds inherent to uncontrolled designs.

This preliminary investigation explicitly functioned as a Phase I proof-of-concept study designed to establish initial feasibility and generate preliminary effect size estimates to inform power analyzes for subsequent controlled trials. Rather than obviating the need for rigorous experimentation, these robust acute effects provide compelling justification for proceeding to Phase II investigations employing experimental controls and longitudinal assessment. This staged approach reflects standard translational research practice wherein initial signal detection precedes mechanistic isolation.

Additionally, the single-session design precludes assessment of effect durability. Whether the substantial psychological improvements observed persist for hours, days, or weeks remains empirically unestablished. Clinical and operational relevance cannot be determined without evidence extending beyond the immediate post-intervention assessment window. Future research must prioritize longitudinal follow-up to distinguish transient mood elevation from durable therapeutic change, as only demonstrated persistence can establish genuine utility for operational populations facing chronic stress demands.

Tolerability was assessed informally through spontaneous adverse event reporting but was not formally quantified using standardized monitoring instruments. No participants discontinued the intervention or reported adverse events during debriefing, suggesting acceptable tolerability for single 23-min sessions. However, a systematic assessment of subjective discomfort, headaches, dizziness, visual disturbances, or other potential side effects was not conducted. Future investigations should incorporate formal adverse event monitoring, particularly for multiple-session protocols or higher-intensity stimulation parameters, as cumulative exposure or increased dosing may reveal tolerability issues not apparent in brief single-session administration. The decision to employ the Muse-S consumer-grade EEG system reflects a deliberate methodological trade-off between ecological validity and neurophysiological measurement fidelity. This device offers substantial practical advantages, including low cost (~$400 versus $10,000+ for research systems), minimal setup requirements, and integration with commercial MindGym deployments. These characteristics positioned it as appropriate for a preliminary, hypothesis-generating investigation. However, these advantages necessitate accepting reduced spatial resolution (4 channels versus 64+ in research systems) and signal quality constraints that fundamentally limit entrainment detection capacity.

Technical constraints inherent to this consumer-grade system severely restricted neurophysiological assessment. The four-channel montage (TP9, AF7, AF8, TP10) provides minimal coverage of occipital and parieto-occipital regions where steady-state visually evoked potentials demonstrate maximal amplitude. Consumer-grade EEG devices lack the sensor density and signal-to-noise ratio necessary to reliably detect frequency-specific entrainment signatures, with additional vulnerability to motion artifacts and environmental interference. The reflective, mylar-lined chamber environment may have created electromagnetic interference conditions, particularly problematic for Bluetooth protocols.

Physiological data attrition was substantial, with 58% of participants (43/74) excluded from heart rate and heart rate variability analyzes due to Bluetooth connectivity failures. These issues were not encountered in prior MindGym investigations employing Wi-Fi-synchronized research-grade systems, suggesting device-environment interactions rather than inherent chamber incompatibility. Consumer-grade photoplethysmography sensors similarly demonstrate reduced precision compared to clinical electrocardiography, particularly for heart rate variability metrics requiring accurate inter-beat interval detection.

Taken together, our null findings regarding neural entrainment and autonomic responses cannot be interpreted as evidence against steady-state visually evoked potential mechanisms or cardiovascular effects. Rather, they reflect instrumentation limitations that render such effects essentially undetectable within our methodological constraints. The mechanistic questions of entrainment and cardiac responses remain inconclusive due to technical limitations rather than absence of effect. This constraint prevented us from addressing a critical gap in AVS research, where psychological improvements are widely assumed to reflect neural entrainment despite scarce empirical verification of such mechanisms^40,42^. A fundamental interpretive constraint stems from using screening PSS scores as the baseline for outcome comparisons. Participants were selected for elevated perceived stress (PSS ≥ 14), creating vulnerability to regression to the mean—the statistical tendency for extreme scores to normalize upon remeasurement independent of intervention. The observed PSS reduction could therefore reflect genuine therapeutic effects, regression artifacts, or both in unknown proportions. The marginally significant negative correlation between baseline PSS and change scores (*r* = −0.24, *p* = 0.047) exemplifies this ambiguity: while potentially indicating differential responsiveness or ceiling effects, this pattern is precisely what regression predicts and cannot be distinguished from statistical artifacts without controlled comparison groups. PIL measures suffer from similar interpretability constraints.

The temporal structure of PSS and PIL assessment introduces additional interpretive ambiguity. While these constructs represent more stable trait-like characteristics than state measures, they can shift acutely following impactful experiences. PSS instructs respondents to report stress “in the last month,” yet our immediate post-intervention assessment may have captured acute mood changes rather than stable shifts in perceived chronic stress. Similarly, PIL’s rapid improvement following a 23-min session could represent state-dependent retrieval biases, demand characteristics, or temporary mood elevation influencing responses to trait-oriented items. Without follow-up assessments at later timepoints, we cannot distinguish durable construct shifts from transient self-report alterations that dissipate as acute psychological states normalize.

By contrast, acute state measures (STAI, POMS, PANAS, FSS, SVS) provide more interpretable evidence. These instruments were administered only on the day of intervention (immediately pre- and post-protocol), were not used as screening criteria, and captured psychological states proximal to treatment rather than days earlier at screening. This temporal structure reduces vulnerability to regression artifacts and minimizes confounding from intervening life events or mood fluctuations across days. Nevertheless, all measures remain vulnerable to demand characteristics, placebo effects, and expectancy in the absence of appropriate control conditions.

Validated burnout inventories (MBI, CBI, OBI, BBI) were administered only at screening and not reassessed post-intervention due to time constraints and prioritization of state-sensitive measures during the limited post-intervention assessment window. Consequently, the inferential link between observed PSS/PIL changes and burnout-related functioning requires direct confirmation through pre-post assessment of validated burnout constructs. We cannot definitively establish whether the substantial improvements in perceived stress and PIL translate to measurable reductions in burnout symptoms without direct measurement. This study relied on recruitment exclusively from the Los Angeles region, which may constrain geographic generalizability. Future work should aim to replicate these findings across multiple sites and geographic contexts. Additionally, participants were drawn from a non-clinical community sample reporting moderate stress levels, which may limit generalizability to clinical populations or those experiencing severe burnout. Because recruitment was community-based rather than occupation-specific, it remains unclear whether the observed effects extend to specific occupational populations or workplace contexts.

This preliminary uncontrolled investigation examined associations between single-session rhythmic AVS (11.5 min) within an immersive reflective chamber (MindGym) and psychological outcomes in a high-stress cohort. Leveraging both alpha and theta-based stimulation protocols, we observed substantial pre-to-post improvements across anxiety, mood, flow states, and vitality, alongside associated enhancements in perceived stress and PIL. The latter constructs demonstrated strong baseline correlations with comprehensive burnout inventories, providing an empirical foundation for interpreting these shifts as potentially burnout-relevant.

No meaningful outcome differences emerged as a function of stimulation frequency. However, exploratory moderation analyzes revealed frequency-specific response patterns wherein baseline mood disturbance predicted differential enhancement of existential purpose in the theta protocol. These patterns suggest potential foundations for precision neurostimulation frameworks guided by momentary affective profiles rather than stable trait characteristics.

Neurophysiological assessment yielded inconclusive results regarding entrainment mechanisms, reflecting acknowledged limitations of consumer-grade instrumentation rather than definitive mechanistic evidence. The minimal occipital coverage and reduced signal fidelity fundamentally constrained detection of steady-state visually evoked potentials, leaving therapeutic mechanisms empirically unresolved.

Critically, this investigation was designed as an exploratory feasibility study rather than a controlled efficacy trial. The absence of control conditions precludes causal attribution. Observed associations may reflect placebo effects, expectancy, demand characteristics, regression artifacts, or genuine intervention-specific mechanisms in unknown proportions. Nevertheless, the study successfully demonstrated initial evidence of feasibility and tolerability, with no adverse events or participant discontinuations.

These preliminary signals—substantial effect magnitudes, differential moderation patterns suggesting mechanistic specificity, and practical deployability—provide compelling justification for proceeding to controlled investigations. Future work incorporating research-grade neurophysiological equipment, validated burnout inventories at multiple timepoints, active and passive control conditions, and longitudinal follow-up will establish whether these associations reflect durable intervention-specific effects. For operational populations experiencing chronic stress exposure and barriers to traditional interventions, this accessible technology offers promising potential for acute stress management, warranting rigorous controlled evaluation to determine its place within the broader landscape of evidence-based therapeutics.

Regarding future directions, controlled experimental designs represent the immediate priority for isolating intervention-specific effects from confounding variables. Waitlist controls would establish baseline spontaneous fluctuation rates in outcome measures, while sham stimulation conditions (e.g., MindGym exposure without stroboscopic protocols, or non-rhythmic light patterns) would permit isolation of expectancy and environmental novelty effects. Random multi-frequency stimulation controls would distinguish frequency-specific therapeutic mechanisms from non-specific AVS effects. Factorial designs systematically varying core components—stroboscopic stimulation (present versus absent), immersive reflective environment (MindGym versus standard room), and their interaction—would enable identification of the active therapeutic ingredient(s) and determine whether effects require full multimodal integration or can be achieved through individual components.

Direct pre-post and follow-up assessment of validated burnout inventories (MBI, CBI, OBI, BBI) represents a critical next step for positioning these interventions within the broader acute intervention landscape. While strong baseline correlations between PSS/PIL and burnout measures (*r* = 0.38 to 0.63; *r* = −0.44 to −0.58) provide an empirical foundation for interpreting current findings as burnout-relevant, demonstrating that acute PSS/PIL improvements translate to measurable reductions in burnout symptom clusters (exhaustion, cynicism, professional inefficacy) requires direct assessment of these validated constructs. This would enable direct comparison with other single-session non-pharmacological interventions and establish whether observed effect sizes represent meaningful shifts on standardized burnout metrics.

Longitudinal protocols would address durability and dose-response questions through systematic manipulation of session frequency, duration, and total exposure. An 8-week protocol where participants complete 1–8 sessions would enable direct comparison with established multi-week interventions such as Mindfulness-Based Stress Reduction. Follow-up measurements at standardized intervals (1-week, 1-month, 3-month, 6-month) would determine whether acute effects persist or require maintenance dosing. Comparative effectiveness trials against pharmacological and psychotherapeutic standards would establish how this non-pharmacological approach, whether delivered as a single session or longitudinally across multiple sessions, compares in efficacy and durability to existing treatment modalities.

Definitive mechanistic understanding requires research-grade neurophysiological assessment while maintaining ecological validity. We recommend a hybrid approach for future investigations: consumer-grade equipment (Muse-S) across all participants to preserve deployment feasibility assessment, supplemented with research-grade neurophysiological monitoring on a representative subsample (30–40% of participants) to ensure adequate data fidelity for mechanistic validation and potential consumer-grade correlates.

High-density EEG arrays (≥32 channels) with occipital coverage and enhanced spatiotemporal resolution could adequately test whether SSVEP entrainment mediates therapeutic effects or whether benefits emerge through alternative neuromodulatory pathways. Concurrent research-grade cardiac monitoring would enable rigorous and more conclusive assessment of autonomic mechanisms. Given substantial data loss encountered with Bluetooth-based systems in the reflective chamber environment, future studies should prioritize hardwired data transmission solutions to maintain transmission in electromagnetically challenging environments.

Future investigations should also directly measure awe experiences as potential mediators linking immersive audiovisual environments to psychological outcomes, examining whether therapeutic effects operate through awe-induced self-transcendence, entrainment-driven oscillatory changes, or their synergistic interaction.

Precision medicine optimization emerges as particularly promising given observed moderation findings. Machine learning algorithms incorporating baseline psychological profiles, particularly mood disturbance indicators that predicted differential responsiveness to theta versus alpha protocols, could enable algorithmic protocol assignment surpassing random allocation efficacy. Variables approaching but not achieving significance after multiple comparison correction warrant inclusion in multivariate prediction models, potentially revealing combinatorial phenotypes optimizing individual treatment matching. This data-driven approach could advance personalized neurostimulation frameworks wherein momentary psychological states guide intervention selection.

Clinical translation demands systematic investigation across high-stress occupational groups exhibiting clinically validated elevated burnout risk, including healthcare workers, first responders (firefighters, emergency medical technicians), emergency physicians, and air traffic controllers. Parallel investigation in clinical diagnostic populations meeting thresholds for anxiety disorders, major depressive disorder, and post-traumatic stress disorder would establish therapeutic range across severity spectra.

The current pilot study was conducted as Phase I of a U.S. Air Force STTR grant; Phase II proposes on-base interventions with Air Force Special Operations Command personnel, providing an ecologically valid assessment of real-world operational deployment. These investigations would incorporate dose-response characterization and comparative effectiveness trials against established standards, with on-site occupational implementation serving as a pragmatic test of field applicability.

Implementation research examining scalability through virtual reality platforms, mobile applications, and home-use devices could democratize access while maintaining therapeutic fidelity—particularly crucial for operational populations requiring immediate, stigma-free interventions. Establishing clinical significance necessitates demonstrating both efficacy under controlled conditions and effectiveness in naturalistic deployment contexts where such interventions would ultimately be utilized.

## Methods

### Participants

A power analysis utilizing Cohen’s conventional frameworks determined that detecting a medium-to-large effect size (*d* = 0.7) with 80% statistical power at α = 0.05 required 34 participants per condition. Anticipating behavioral exclusions and technical complications inherent in complex neurotechnology protocols, a total of 74 individuals from the greater Los Angeles area (41 females; age range 20–69 years; *μ* = 39.69, *σ* = 13.35) participated in the study, of which 67 were employed either full-time or part-time.

Five participants were excluded from analyzes of the psychological measures analysis due to technical issues (e.g., lack of audio; *N* = 2), behavioral errors (e.g., facing the wrong direction during the experience; *N* = 1), or incomplete questionnaire data (*N* = 2). As a result, a total of 69 participants were included in the analyzes of psychological measures analysis (38 females; age range 20–69 years; *μ* = 40.09, *σ* = 13.67)

Forty-three participants were excluded from the physiological analysis due to a connection error that prevented data writing, resulting in a total of 31 participants included in the statistical analyzes related to physiology (20 females; age range 21–63 years; *μ* = 40.74, *σ* = 12.67).

Eight participants were excluded from the EEG analysis due to a connection error that prevented data from being saved and technical data storage error, resulting in a total of 66 participants included in the statistical analysis (37 females; age range 20–69 years; *μ* = 40.33, *σ* = 13.14).

Ten participants were excluded from the EEG/Psychological Measures Moderation analysis due to the above issues with EEG analysis (*N* = 8) and incomplete questionnaire data (*N* = 2), resulting in a total of 64 participants included in the EEG/Psychological Measure moderation analysis (35 females; age range 21–63 years; *μ* = 39.69; *σ* = 13.45).

**Fig. 4:**
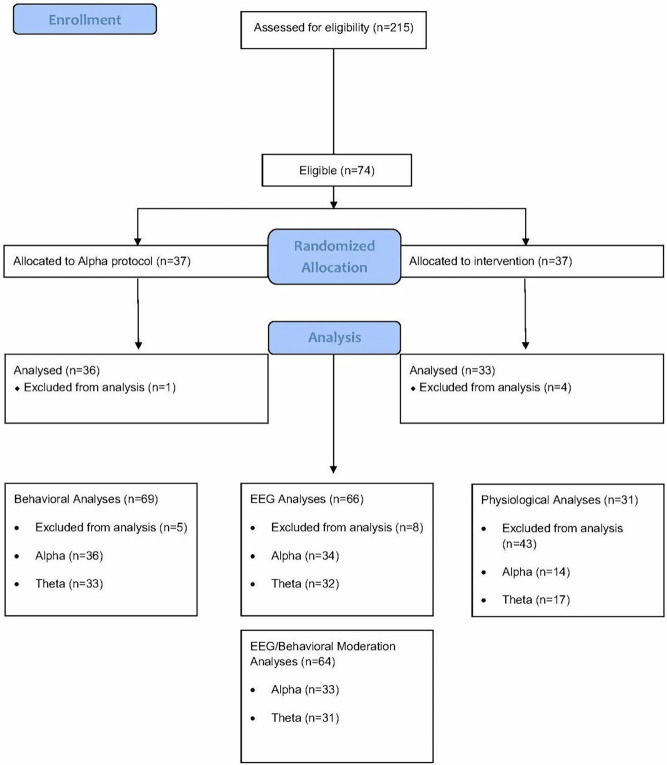
Consolidated Standards of Reporting Trials (CONSORT)-style flow diagram. CONSORT-style flow diagram depicting movement of participants and data through the experiment.

All participants were randomly assigned to one of two groups (Table 7). Participant flow through enrollment, allocation, exclusions and analysis (Fig. 4).

**Table 7:** Participant characteristics for measures by group and analysis type

Measure	Group	*N*	Gender (M/F)	Age range	*μ*	*σ*
Psychological measures						
	Alpha	36	16 Male, 20 Female	20–69	39.56	14.33
	Theta	33	15 Male, 18 Female	21–63	40.67	13.12
Physiology						
	Alpha	14	1 Male, 13 Female	21–63	40	14.63
	Theta	17	10 Male, 7 Female	21–61	41.35	11.24
EEG						
	Alpha	34	15 Male, 19 Female	20–69	38.97	13.80
	Theta	32	14 Male, 18 Female	21–63	41.78	12.45
EEG /Psychological measure moderation						
	Alpha	33	15 Male, 18 Female	20–69	39.18	13.95
	Theta	31	14 Male; 17 Female	21–63	41.84	12.65

Age is reported in years. Μ and σ represent the mean and standard deviation of age, respectively.

*F* female, *M* male.

Subjects were assigned to one of two groups using a MATLAB-based randomization algorithm that maintained gender balance, with recruitment ceasing for each gender category upon reaching the predetermined quota. Participants were randomly assigned to one of two stimuli: Alpha or Theta.

Participants were recruited through multiple channels—newsletters to previous research participants and targeted social media advertisements (Facebook, Craigslist, Instagram)—within a 50-mile radius of Santa Monica, California (July 22, 2024–March 4, 2025). Compensation comprised $30 per hour (cash or Venmo), calculated from check-in to departure and rounded to the nearest quarter-hour increment, with validated parking provided.

Participants were eligible for the study if they met all of the following criteria: (1) had no hairstyles such as braids, cornrows, dreadlocks, weaves, or extensions that could interfere with scalp sensor contact; (2) were not pregnant or possibly pregnant; (3) had no history of neurological conditions including epilepsy, seizures, or stroke; (4) had an absence of psychiatric disorders such as bipolar disorder or schizophrenia; (5) had no history of migraines; (6) had no photosensitivity or photophobia; (7) were free from eye conditions including cataracts, corneal abrasions, keratitis, or uveitis; (8) had no hearing impairments; (9) had no history of claustrophobia; (10) had no history of vertigo or motion sickness; (11) had no fear of darkness; (12) had normal or corrected-to-normal vision; (13) had sufficient mobility to participate without wheelchairs, walkers, or canes; and (14) were not currently taking medications affecting the central nervous system (including psychostimulants, antidepressants, or antipsychotics) or specific medications known to affect sensory perception (including but not limited to regular doses of NSAIDs, Dilantin, Methotrexate, tetracycline antibiotics, Digoxin, Amiodarone, Atropine, phenothiazine antipsychotics, H2 blockers, Fingolimod, aminoglycoside antibiotics, loop diuretics, and certain chemotherapeutics); (15) had not selected the response option indicating they had “seriously thought of it as a way out” on the suicide-related item from the PIL^46^; and (16) received a score of 14 or higher on the PSS^45^, indicating moderate to high perceived stress levels.

All potential participants completed a comprehensive online screening questionnaire via Google Forms to verify eligibility before study enrollment. This preliminary assessment verified that all participants met the established inclusion and exclusion parameters prior to beginning the research protocol.

All recruitment and testing procedures were reviewed and approved by the Advarra Institutional Review Board (Columbia, MD) prior to the start of participant enrollment (Pro00079710). In accordance with ethical guidelines and legal obligations, all participants provided written informed consent via a document hosted on DropboxSign. Participants were given sufficient time to ask questions and receive clarification from the Principal Investigator or study staff. The consent process included the California Experimental Research Bill of Rights, as required by Health and Safety Code Section 24172. The study was conducted in alignment with the principles of the Declaration of Helsinki. In addition, all laboratory staff held current certifications in Good Clinical Practice and Human Research Participant Protection, completed through accredited online training programs. The clinical trial number is not applicable.

Given the sensitivity of the study population, research staff were trained to follow a safety protocol in the event of participant suicidality. A licensed physician was on call during all sessions and was to be contacted immediately for clinical assessment if a participant exhibited urgent signs (e.g., suicidal intent or severe distress). If further action was deemed necessary, staff were instructed to call 911 and coordinate emergency transport to one of two pre-identified hospitals within 1.5 miles of the testing site, with staff instructed to remain with the participant until help arrived and report the incident to the Principal Investigator, IRB, and study sponsors in accordance with adverse event procedures. However, no such adverse events occurred.

### Materials

All participants sat on an OMEGA Gaming Chair (SecretLab, Inc.), wore noise-canceling, over-ear Bluetooth QuietComfort 45 headphones (Bose Corporation), and completed the psychological questionnaires (administered through Google Forms) on a 27-inch 2022 iMac (Apple, Inc.), using a wireless keyboard and mouse, regardless of group.

MindGym (Lumena, Inc.) is a 7’ cubic enclosure, isotropic MindGym lined with reflective mylar on its interior walls, while its floor and ceiling are equipped with mirrors. MindGym incorporates WS2815 LEDs, with 121 pixels per edge between the vertices, totaling 1452 pixels across 12 edges. It utilizes a SMD5050 RGB LED chip, offering RGB color with 256 grayscale levels and an output of 990-1080 lumens per meter. The LEDs are operated at less than half of their maximum capacity. Additionally, the system features a color temperature of 5500 K. MindGym has been used previously by our group as an anxiolytic experiential technology ^44^.

All participants wore the Muse-S (InteraXon, Inc.), a consumer-grade EEG, multi-sensory headband featuring four EEG sensors (two on the forehead [AF7, AF8]; two behind the ears [TP9, TP10]; reference at FPz) with a sampling rate of 256 Hz, along with Photoplethysmography (PPG). The Muse-S was selected because it ships as the integrated biosensor with the commercial Lumena MindGym system; this pilot investigation aimed to evaluate the Muse’s viability for real-time entrainment detection measurement in deployed operational settings, despite known limitations in spatial resolution and signal-to-noise ratio compared to research-grade systems. Additionally, the reflective, mylar-lined chamber may have contributed to Bluetooth connectivity issues and data loss not encountered with Wi-Fi-based electrocardiogram (ECG) systems in prior work. The Muse-S connected to the “Muse: Meditation & Sleep” app on a 10.9-inch 2022 iPad Air (Apple, Inc.) to initialize satisfactory signal quality via the built-in quality check.

Neuropype (Intheon, San Diego, CA) was leveraged to receive raw EEG data via a lab-streaming layer stream. The pipeline applies minimal preprocessing: timestamp dejittering to create evenly spaced samples across channels, followed by a FIR bandpass filter with edge frequencies at 0.5, 1, 65, and 70 Hz to eliminate typical noise artifacts.

Psychological questionnaires were administered during screening, pre and post experiment to gather trait and state information (See Table 8).

**Table 8:** Psychological questionnaires denoted by measure and timepoint

Measure	Time	Questionnaire	Description
Burnout	Screening	Copenhagen Burnout Inventory (CBI)	The Copenhagen Burnout Inventory (CBI) is a 19-item self reported measure of burnout. It contains three sub-scales measuring personal burnout, work-related burnout, and client-related burnout^74^.
		Oldenburg Burnout Inventory (OBI)	The 16-item Oldenburg Burnout Inventory (OLBI) was originally constructed and validated among different German occupational groups^75,76^. It assesses the two core dimensions of burnout: exhaustion and disengagement (from work). Exhaustion is defined as a consequence of intensive physical, affective, and cognitive strain (i. e., as a long-term consequence of prolonged exposure to certain job demands). The 8 items of the Exhaustion subscale are generic, and refer to general feelings of emptiness, overtaxing from work, a strong need for rest, and a state of physical exhaustion. The 8 items on the Disengagement subscale refer to distancing oneself from the object and the content of one’s work and to negative, cynical attitudes and behaviors toward one’s work in general.
		Maslach Burnout Inventory (MBI)	The Maslach Burnout Inventory (MBI) is a 16-item psychological assessment tool designed to measure burnout levels in individuals, particularly in professional settings. It includes various versions, such as the MBI-General Survey (MBI-GS) and the MBI-Human Services Survey (MBI-HSS). The MBI assesses three key dimensions: Emotional Exhaustion, Cynicism, and Professional Efficacy, helping to identify burnout symptoms and their impact on work performance^77^.
		Bergen Burnout Inventory (BBI)	The Bergen Burnout Inventory (BBI) consists of three subscales: (1) exhaustion at work; (2) cynicism towards the meaning of work; and (3) sense of inadequacy at work^78^.
Trait	Pre	Ten-Item Personality Inventory (TIPI)	Ten items to measure the Big Five personality dimensions: openness to experience, conscientiousness, extraversion, agreeableness, and neuroticism^79^.
		Dispositional Hope Scale (DHS)	Twelve items to measure the feeling of hope on a Likert scale from 1 (definitely false) to 4 (definitely true), divided among three subscales: pathways, agency, and negative filler items that aren’t related to hope^80^.
		General Self-Efficacy Scale (GSES)	Ten items to measure self-efficacy in a professional setting on a Likert scale from 1 (not at all true) to 4 (exactly true). Also measures if self-efficacy is a predictor of burnout and engagement^81^.
		Multidimensional Health Locus of Control (MHLC)	Eighteen items to assess the extent to which individuals believe they have control over their health, divided among three subscales: internality, powerful others externality, and chance externality^82^.
		Connor-Davidson Resilience Scale (CD-RISC-10)	Ten items to measure resilience (the ability to adapt from stress, trauma, and adversity), on a Likert scale from 0 (not true at all) to 4 (nearly true all the time), using five subscales: personal competence, acceptance of change and secure relationships, trust/tolerance/strengthening effects of stress, control, and spiritual influences^83^.
		Dispositional Resilience ‘Hardiness’ Scale (HARDY)	Forty-five items to measure hardiness–a trait associated with resilience and stress tolerance–using three subscales: communication scale, challenge scale, and control scale^84^.
		State-Trait Anxiety Inventory (STAI)	A measure of state anxiety (temporary/situational) and trait anxiety (enduring propensity) (Spielberger et al., 1983).
State	Pre & post	Profile of Mood States (POMS)	A measure of current mood states across affective dimensions like anger, confusion, depression, fatigue, tension, and vigor^85^.
		Anxiety-Emoji-based Scale	A single-item measure using five animated emoji images, ranging from very happy to very sad, to represent the respondent’s current level of anxiety^86^.
		Subjective Vitality Scale (SVS)	Six items to assess subjective vitality via self-reported positive mental energy and alertness, using a Likert scale from 1 (not at all true) to 7 (very true)^52^.
		Positive and Negative Affect Schedule (PANAS)	Twenty items used to measure the presence and intensity of emotions, using two subscales: positive affect and negative affect^87^.
		Flow State Scale (FFS) (short version)	Nine items to assess flow experience using two subscales: absorption by activity factor and fluency of performance factor^88^.
		State-Trait Anxiety Inventory (STAI)	A measure of state anxiety (temporary/situational) and trait anxiety (enduring propensity)^89^.
Outcome	Screening & post	Purpose in Life Test (PIL)	Twenty items to measure one’s sense of perceived purpose or meaning in life. Respondents rate their agreement with statements about their life experience on a scale from 1 (feelings of no purpose) to 5 (the greatest feelings of purpose)^46^.
		Perceived Stress Scale (PSS)	Ten items to measure how different situations affect feelings of stress, measured on a scale from 0 (never) to 4 (very often)^45^.
	Post	Emotional Breakthrough Inventory (EBI)	Six items to assess acute emotional breakthroughs using a scale from 0 (no, not more than usual) to 10 (yes, entirely or completely)^90^.
		Toronto Mindfulness Scale (TMS)	Thirteen items to measure state mindfulness (administered post-meditation), differentiating between reflective awareness and ruminative attention, using two subscales: curiosity (gauging interest in one’s experiences) and decentering (reflecting the ability to view thoughts and feelings as transient mental events)^91^.

### Procedure and stimuli

**Fig. 5:**
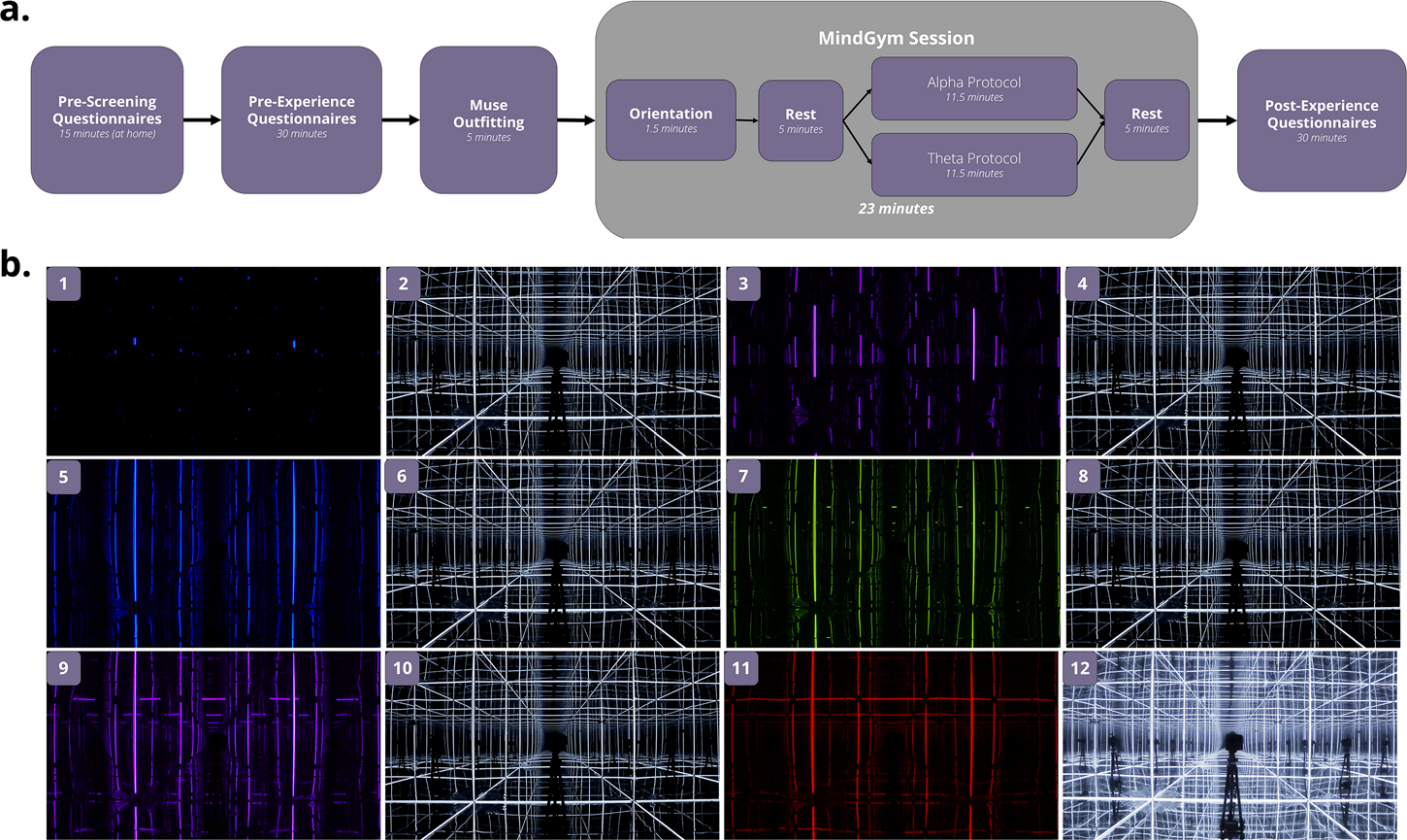
Procedure overview and sequential progression of stroboscopic audiovisual entrainment protocol within MindGym chamber. **a** Total participation time was approximately 103 min. Participants first completed pre-screening questionnaires at home (~15 min), with an average interval of 48 days (SD = 65) before the in-laboratory visit. In the lab, participants completed pre-experience questionnaires (~30 min), were fitted with the Muse EEG headband including impedance verification (~5 min), completed the MindGym session (23 min; see **b** for session structure), and concluded with post-experience questionnaires (~30 min). **b** Panels 1–12 depict the 11.5-min stroboscopic portion of the 23-min MindGym session. Odd-numbered panels represent eyes-open periods featuring progressive LED activation—beginning with minimal vertical coverage (Panel 1; 2 min), expanding vertically (Panels 3, 5; 30 s each), introducing horizontal arrays with lateral sweeping (Panel 7; 30 s), then expanding horizontal coverage (Panels 9, 11; 30 s and 1 min, respectively). Eyes-open periods cycled through blue, purple, green, and red; representative colors shown. Even-numbered panels represent eyes-closed periods with full white light activation across all vertices (Panels 2, 4, 6, 8, 10; 30 s each), culminating in progressive luminosity intensification (Panel 12; 4 min). Not pictured: verbal orientation (1.5 min), pre-rest (5 min, eyes-closed, dark), and post-rest (5 min, eyes-closed, dark). See a recording within the MindGym of the Theta stroboscopic stimulation [https://youtu.be/-6N9RBqQ4xI] for complete audiovisual experience.

Total participation time was approximately 103 min, distributed across remote and in-laboratory components (Fig. 5A). Participants first completed pre-screening questionnaires at home (~15 min), with an average interval of 48 days (*SD* = 65) before the in-laboratory visit. The laboratory session comprised pre-experience questionnaires (~30 min), EEG headband fitting (~5 min), the MindGym session (23 min; Fig. 5B), and post-experience questionnaires (~30 min).

Before each session, all participants signed eConsent forms (DropboxSign). Upon arrival, participants were seated on an office chair facing a 35.5” × 55” desk and given an overview of the session structure. They were informed that their “experience” would involve sitting for 23 min in an immersive audiovisual environment.

Participants first completed pre-experience questionnaires on Google Forms, including the TIPI, DHS, GSES, MHLC, CD-RISC-10, HARDY, STAI, GA-VAS, POMS, SVS, PANAS, FFS-short version, and an Anxiety Emoji-based scale (~30 min). Participants were instructed to silence their phones and remove smartwatches or electronic wristbands that emit light.

Using a cotton pad and 91% isopropyl alcohol, the experimenter cleansed the participant’s forehead and areas behind the ears before outfitting the participant with the Muse-S headband, ensuring a snug fit to optimize sensor connectivity. Signal quality was verified using the Muse: Meditation & Sleep app, which displayed visual indicators for each sensor (green signifying good connection; red denoting no connection). The experimenter adjusted the headband as needed until all sensors displayed green indicators. Additionally, filtered data from Neuropype (see “Materials”) was displayed in a live time-series plot showing all EEG channels. A research assistant visually inspected the plot to confirm that each channel delivered a stable and discernible signal, checking for possible obstructions such as hair or clothing interfering with sensor contact until the signal was visually acceptable.

Participants were led into MindGym, seated centrally, provided noise-canceling headphones, and shown how to exit if they chose to end the experience early; otherwise, the experimenter would return to assist them once the experience ended. The experience began within 30 s of the MindGym door closing.

The standardized 23-min protocol commenced with brief verbal orientation (1.5 min) via a pre-recorded audio track which instructed participants to face the wall 90 degrees to their left relative to the entrance, sit comfortably, breathe normally, and follow auditory prompts for opening and closing their eyes throughout the experience. Orientation was immediately followed by a 5-min pre-rest baseline period (eyes closed, dark, no stimulation) to establish resting state conditions. The subsequent 11.5-min stroboscopic sequence comprised 12 stages alternating between eyes-open and eyes-closed conditions (Fig. 2B). Participants underwent one of two audiovisual entrainment protocols—theta (4–7 Hz) or alpha (9–11 Hz). The experimental conditions maintained identical temporal sequences, visual progression patterns, and instructional frameworks, differing exclusively in the frequency parameters of both visual and audio stimulation. As part of the audiovisual sequence, synchronized binaural audio tracks reinforced targeted theta or alpha frequency bands, intended to encourage multisensory entrainment. A video demonstration of the theta protocol’s active stimulation phase (excluding pre- and post-intervention resting periods) is available at https://www.youtube.com/watch?v=-6N9RBqQ4xI.

Eyes-open stages (Stages 1, 3, 5, 7, 9, 11) featured progressive LED activation with chromatic cycling through blue, purple, green, and red spectral illumination. Stage 1 (2 min) initiated vertical LED activation at minimal coverage. Stages 3 and 5 (30 s each) progressively expanded vertical coverage. Stage 7 (30 s) achieved full vertical activation while introducing horizontal LED arrays with lateral sweeping motion. Stage 9 (30 s) maintained full vertical activation while initiating center horizontal LED expansion. Stage 11 (1 min) culminated in complete vertical and horizontal activation across all vertices (Fig. 5).

Eyes-closed stages (Stages 2, 4, 6, 8, 10) employed full-spectrum white light activation across all vertices (30 s each). The final eyes-closed stage (Stage 12; 4 min) implemented progressive luminosity intensification, systematically increasing brightness to create an intensified sensory finale. The protocol concluded with a 5-min post rest period (eyes closed, dark, no stimulation).

After the experience, the Muse-S headband was removed and participants were given the opportunity to use the restroom if needed before completing post-experience questionnaires, including the STAI, GA-VAS, POMS, SVS, PANAS, FFS-short version, EBI, PIL, PSS, TMS, and an Anxiety Emoji-based scale (~30 min). Finally, participants were remunerated, their parking validated, and dismissed.

### Psychological measure analyses

The primary dependent variables in this study were scores on self-report psychological measures administered pre- and post-intervention. State measures were collected on the day of the intervention at two timepoints—immediately before (pre) and immediately after (post) the AVS protocol—and included: anxiety measures—State-Trait Anxiety Inventory (STAI-State) and Anxiety Emoji Scale; mood measures—POMS subscales (Depression, Tension, Anger, Fatigue, Confusion) and total score, and Positive and Negative Affect Schedule (PANAS); flow measures—FSS subscales (Absorption by Activity, Fluency of Performance) and total score; and vitality measure—SVS. Outcome measures were collected at two timepoints—screening (serving as pre-intervention baseline) and post-intervention—and included: PIL and PSS. PSS was prioritized as the primary eligibility criterion (PSS ≥ 14) because it most directly captures felt affective experience of stress burden, while comprehensive burnout inventories (BBI, MBI, CBI, OBI) were conceptualized as indexing downstream consequences of chronic stress exposure. This temporal assessment structure reflects a deliberate prioritization: screening protocols were comprehensive to fully characterize the sample’s burnout-related functioning, whereas post-intervention assessments prioritized state-sensitive measures capable of detecting acute psychological changes while limiting participant burden during the brief post-intervention window. The other measures were selected for pre-to-post assessment due to their hypothesized sensitivity to acute psychological interventions and direct relevance to stress mitigation and subjective well-being. Post-intervention-only measures included: EBI and TMS, which were administered only after the intervention to assess acute phenomenological experiences. Additional screening-only measures (burnout inventories: BBI, MBI, CBI, OBI) were collected solely at screening to characterize the sample and to validate PSS and PIL as burnout-related indicators but were not re-administered post-intervention.

The primary independent variables were: condition (between-subjects factor), comparing alpha neurostimulation (9–11 Hz) with theta neurostimulation (4–7 Hz); deltas between Pre-intervention and Post-intervention assessments; and Pre-intervention versus Post-intervention assessments.

Prior to conducting the ANOVAs, the data were assessed for violations of parametric assumptions, including normality using Shapiro–Wilk tests and homogeneity of variance using Levene’s test. In cases where these assumptions were substantially violated, equivalent nonparametric alternatives (e.g., Wilcoxon signed-rank tests for within-subject comparisons and Mann-Whitney U tests for between-subject comparisons) were utilized.

To examine whether individual differences moderated the effects of the interventions, linear regression analyzes were conducted. In these analyzes, changes in variables (post-pre difference scores) and also outcome variables were regressed on condition (alpha vs. theta), potential moderator variables (TIPI, DHS, GSES, MHLC, CD-RISC-10, HARDY, etc.), and their interaction terms. Significant interaction terms would indicate that the effectiveness of the interventions varied as a function of individual differences on the trait measures. EEG baseline measures were also examined as potential moderators of intervention effects. Linear regression analyzes were conducted with changes in outcome variables regressed on condition (alpha vs. theta), baseline EEG parameters, and their interaction terms. Significant interactions would indicate that baseline brain activity patterns influenced differential responsiveness to alpha versus theta interventions.

A series of 2 × 2 mixed-design repeated measures ANOVAs was conducted to evaluate the effects of the interventions on all dependent variables. Each ANOVA included condition (alpha vs. theta) as a between-subjects factor and time (pre-intervention vs. post-intervention) as a within-subjects factor. The main effects of condition and time, as well as their interaction, were examined for each dependent variable. Post hoc analyzes were conducted on initial significant results. Effect sizes were reported using generalized eta squared (*η²*_*G*_) for the mixed design ANOVAs, and Cohen’s *d* (*d*) for post hoc analyzes. For *d*, values of 0.2, 0.5, and 0.8 indicated small, medium, and large effects, respectively^65^. For *η²*_*G*_, values of 0.02, 0.13, and 0.26 indicated small, medium, and large effects, respectively^66^.

Mediation analyzes were conducted to explore potential mechanisms underlying the observed effects. Specifically, we examined whether changes in state anxiety and mood states mediated the relationship between the interventions and changes in outcome variables. These analyzes followed the bootstrapping approach recommended by Preacher and Hayes^67^ using 5000 bootstrap samples to estimate indirect effects and their 95% confidence intervals.

### EEG analyses

EEG preprocessing began with timestamp dejittering to ensure evenly spaced samples across all channels, followed by the removal of bad channels identified during a 60-s calibration period based on signal variance. A FIR highpass filter (0.5–1 Hz passband, 80 dB stopband attenuation) was then applied to eliminate slow drifts and baseline shifts while preserving low-frequency neural activity. After filtering, artifact removal was performed by excluding data segments that exceed predefined thresholds. A FIR notch filter (55–65 Hz) was applied to suppress 60 Hz line noise common in lab environments, and a FIR lowpass filter (95–100 Hz) was used to attenuate high-frequency muscle and equipment artifacts. Channels previously removed due to poor signal quality are then re-interpolated using spatial information from neighboring electrodes, restoring full topographic coverage for downstream analysis.

### Multiple comparison corrections

Multiple comparison corrections were applied according to each analysis’s statistical structure and research objectives. Primary hypotheses regarding main effects of time and condition underwent Bonferroni correction (*α* = 0.05) as confirmatory analyzes requiring stringent Type I error control.

Moderation analyzes, designated as exploratory for future hypothesis generation, were corrected using the Benjamini–Hochberg FDR (*α* = 0.05) applied separately within five conceptually distinct families: (1) state moderation—pre-intervention state measures moderating changes in corresponding state variables (15 tests); (2) trait moderation—personality traits moderating state changes (120 tests); (3) state–outcome moderation—pre-intervention states moderating outcome changes (60 tests); (4) trait–outcome moderation—personality traits moderating outcome changes (32 tests); and (5) EEG moderation—baseline brain activity moderating intervention effectiveness on psychological measure outcomes (64 tests). Within each family, FDR was applied exclusively to interaction effects, targeting exploratory moderation findings.

This family-wise FDR framework increased sensitivity to potential moderator effects while maintaining principled control over false discoveries within each conceptual domain, aligning with established approaches for exploratory analyzes aimed at identifying predictors of treatment response^68,69^.

## Supplementary information

Supplementary Materials

## Data Availability

The psychological questionnaire data, EEG data, and physiological data (heart rate and heart rate variability) that support the findings of this study are openly available on the Open Science Framework (OSF) at https://osf.io/3sjeu. The audiovisual stimulation protocols (alpha and theta conditions) utilized proprietary MindGym hardware and software systems developed by Lumena, Inc. While the general parameters of these protocols are described in detail in the Methods section, the specific control programs and LED sequences are proprietary to Lumena, Inc. and are not publicly available. Researchers interested in replicating these protocols using the MindGym platform should contact Lumena, Inc. directly. Raw consent forms and participant identification information are not publicly available to protect participant privacy and confidentiality in accordance with IRB approval (Advarra IRB Pro00079710) and HIPAA regulations. De-identified data are available as described above. Requests for additional information or materials should be directed to the corresponding author.

## Code availability

Code used to generate supplementary figures and perform secondary analyses is available at the repository https://github.com/Institute-for-Advanced-Consciousness/P006. All first pass analyses were conducted in JASP 0.19.3^70^. FDR corrections for moderation analyses were conducted in Python 3.12.2 using the statsmodel module version 0.14.0 leveraging the Benjamini–Hochberg method^71^. Post-hoc EEG moderation of psychological measures were conducted in Python 3.12.2 using the statsmodel module version 0.14.0^71^ and supplementary figures were made using matplotlib version 3.10.0^72^ and seaborn version 0.13.2^73^.

## References

[1] Schneiderman, N., Ironson, G. & Siegel, S. D. Stress and health: psychological, behavioral, and biological determinants. *Annu. Rev. Clin. Psychol.* **1**, 607–628 (2005). 17716101 10.1146/annurev.clinpsy.1.102803.144141 PMC2568977

[2] Daly, M. & Macchia, L. Global trends in emotional distress. *Proc. Natl. Acad. Sci. USA* **120**, e2216207120 (2023). 36972447 10.1073/pnas.2216207120 PMC10083620

[3] Piao, X., Xie, J. & Managi, S. Continuous worsening of population emotional stress globally: universality and variations. *BMC Public Health* **24**, 3576 (2024). 39716139 10.1186/s12889-024-20961-4 PMC11668040

[4] Fioroni, S. & Foy, D. Americans Sleeping Less, More Stressed. Gallup.com https://news.gallup.com/poll/642704/americans-sleeping-less-stressed.aspx (2024).

[5] O’Connor, D. B., Gartland, N. & O’Connor, R. C. Stress, cortisol and suicide risk. *Int. Rev. Neurobiol.* **152**, 101–130 (2020). 32450993 10.1016/bs.irn.2019.11.006

[6] Sinha, R. Chronic stress, drug use, and vulnerability to addiction. *Ann. N. Y. Acad. Sci.* **1141**, 105–130 (2008). 18991954 10.1196/annals.1441.030 PMC2732004

[7] Arnsten, A. F. T. Stress signalling pathways that impair prefrontal cortex structure and function. *Nat. Rev. Neurosci.* **10**, 410–422 (2009). 19455173 10.1038/nrn2648 PMC2907136

[8] Liston, C., McEwen, B. S. & Casey, B. J. Psychosocial stress reversibly disrupts prefrontal processing and attentional control. *Proc. Natl. Acad. Sci. USA* **106**, 912–917 (2009). 19139412 10.1073/pnas.0807041106 PMC2621252

[9] Shields, G. S., Sazma, M. A. & Yonelinas, A. P. The effects of acute stress on core executive functions: A meta-analysis and comparison with cortisol. *Neurosci. Biobehav. Rev.* **68**, 651–668 (2016). 27371161 10.1016/j.neubiorev.2016.06.038 PMC5003767

[10] Ulrich-Lai, Y. M. & Herman, J. P. Neural regulation of endocrine and autonomic stress responses. *Nat. Rev. Neurosci.* **10**, 397–409 (2009). 19469025 10.1038/nrn2647 PMC4240627

[11] de Kloet, E. R., Joëls, M. & Holsboer, F. Stress and the brain: from adaptation to disease. *Nat. Rev. Neurosci.* **6**, 463–475 (2005). 15891777 10.1038/nrn1683

[12] Lupien, S. J., McEwen, B. S., Gunnar, M. R. & Heim, C. Effects of stress throughout the lifespan on the brain, behaviour and cognition. *Nat. Rev. Neurosci.* **10**, 434–445 (2009). 19401723 10.1038/nrn2639

[13] McEwen, B. S., Nasca, C. & Gray, J. D. Stress effects on neuronal structure: hippocampus, amygdala, and prefrontal cortex. *Neuropsychopharmacology* **41**, 3–23 (2016). 26076834 10.1038/npp.2015.171 PMC4677120

[14] Sheline, Y. I., Wang, P. W., Gado, M. H., Csernansky, J. G. & Vannier, M. W. Hippocampal atrophy in recurrent major depression. *Proc. Natl. Acad. Sci. USA* **93**, 3908–3913 (1996). 8632988 10.1073/pnas.93.9.3908 PMC39458

[15] Buckley, T. M. & Schatzberg, A. F. On the interactions of the hypothalamic-pituitary-adrenal (HPA) axis and sleep: normal HPA axis activity and circadian rhythm, exemplary sleep disorders. *J. Clin. Endocrinol. Metab.* **90**, 3106–3114 (2005). 15728214 10.1210/jc.2004-1056

[16] Coryell, W., Young, E. & Carroll, B. Hyperactivity of the hypothalamic–pituitary–adrenal axis and mortality in major depressive disorder. *Psychiatry Res.* **142**, 99–104 (2006). 16631257 10.1016/j.psychres.2005.08.009

[17] Sekel, N. M., et al. Military tactical adaptive decision making during simulated military operational stress is influenced by personality, resilience, aerobic fitness, and neurocognitive function. *Frontiers in Psychology* **14**, 1102425 (2023). 10.3389/fpsyg.2023.1102425 PMC9944034 36844343

[18] Hosseini, S. M., Hesam, S. & Hosseini, S. A. Burnout among military personnel: a systematic review. *Iran. J. Psychiatry* **18**, 213–236 (2023). 37383961 10.18502/ijps.v18i2.12371 PMC10293693

[19] Li, L. Z. et al. Nurse burnout and patient safety, satisfaction, and quality of care: a systematic review and meta-analysis. *JAMA Netw. Open* **7**, e2443059 (2024). 39499515 10.1001/jamanetworkopen.2024.43059 PMC11539016

[20] Rotenstein, L. S. et al. Prevalence of burnout among physicians: a systematic review. *JAMA* **320**, 1131–1150 (2018). 30326495 10.1001/jama.2018.12777 PMC6233645

[21] Igboanugo, S., Bigelow, P. L. & Mielke, J. G. Health outcomes of psychosocial stress within firefighters: a systematic review of the research landscape. *J. Occup. Health* **63**, e12219 (2021). 33780075 10.1002/1348-9585.12219 PMC8006668

[22] Carhart-Harris, R. L. et al. Psilocybin for treatment-resistant depression: fMRI-measured brain mechanisms. *Sci. Rep.* **7**, 13187 (2017). 29030624 10.1038/s41598-017-13282-7 PMC5640601

[23] Billioti De Gage, S. et al. Benzodiazepine use and risk of dementia: prospective population based study. *BMJ* **345**, e6231–e6231 (2012). 23045258 10.1136/bmj.e6231 PMC3460255

[24] Baldwin, D. S. et al. Benzodiazepines: risks and benefits. a reconsideration. *J. Psychopharmacol.* **27**, 967–971 (2013). 24067791 10.1177/0269881113503509

[25] Safron, A., Juliani, A., Reggente, N., Klimaj, V. & Johnson, M. On the varieties of conscious experiences: Altered Beliefs Under Psychedelics (ALBUS). *Neurosci. Conscious*. **2025**, niae038 (2025). 10.1093/nc/niae038 PMC11823823 39949786

[26] O’Donnell, K. C., Mennenga, S. E. & Bogenschutz, M. P. Psilocybin for depression: considerations for clinical trial design. *J. Psychedelic Stud.* **3**, 269–279 (2019).

[27] Labelle, L. E., Campbell, T. S. & Carlson, L. E. Mindfulness-based stress reduction in oncology: evaluating mindfulness and rumination as mediators of change in depressive symptoms. *Mindfulness* **1**, 28–40 (2010).

[28] Crane, C. & Williams, J. M. G. Factors associated with attrition from mindfulness-based cognitive therapy in patients with a history of suicidal depression. *Mindfulness* **1**, 10–20 (2010). 21125023 10.1007/s12671-010-0003-8 PMC2987524

[29] Hilton, L. et al. Mindfulness meditation for chronic pain: systematic review and meta-analysis. *Ann. Behav. Med.* **51**, 199–213 (2017). 27658913 10.1007/s12160-016-9844-2 PMC5368208

[30] MacKenzie, M., Abbott, K. & Kocovski, N. Mindfulness-based cognitive therapy in patients with depression: current perspectives. *Neuropsychiatr. Dis. Treat.* **14**, 1599–1605 (2018). 29950842 10.2147/NDT.S160761 PMC6018485

[31] Brandmeyer, T., Simmons, R. & Reggente, N. *Navigating the ‘Zen Zeitgeist’: The Potential of Personalized Neurofeedback for Meditation*. 10.31234/osf.io/x23me (2023).

[32] Lomas, T., Ivtzan, I. & Fu, C. H. Y. A systematic review of the neurophysiology of mindfulness on EEG oscillations. *Neurosci. Biobehav. Rev.* **57**, 401–410 (2015). 26441373 10.1016/j.neubiorev.2015.09.018

[33] Lam, S. U., Kirvin-Quamme, A. & Goldberg, S. B. Overall and differential attrition in mindfulness-based interventions: a meta-analysis. *Mindfulness* **13**, 2676–2690 (2022). 36506616 10.1007/s12671-022-01970-z PMC9728563

[34] Klimesch, W. EEG alpha and theta oscillations reflect cognitive and memory performance: a review and analysis. *Brain Res. Rev.* **29**, 169–195 (1999). 10209231 10.1016/s0165-0173(98)00056-3

[35] Başar, E., Başar-Eroglu, C., Karakaş, S. & Schürmann, M. Gamma, alpha, delta, and theta oscillations govern cognitive processes. *Int. J. Psychophysiol.* **39**, 241–248 (2001). 11163901 10.1016/s0167-8760(00)00145-8

[36] Golonka, K., Gawlowska, M., Mojsa-Kaja, J. & Marek, T. Psychophysiological characteristics of burnout syndrome: resting-state EEG analysis. *BioMed Res. Int.* **2019**, 3764354 (2019). 31467886 10.1155/2019/3764354 PMC6701350

[37] Leuchter, A. F., Cook, I. A., Hunter, A. M., Cai, C. & Horvath, S. Resting-state quantitative electroencephalography reveals increased neurophysiologic connectivity in depression. *PLoS ONE* **7**, e32508 (2012). 22384265 10.1371/journal.pone.0032508 PMC3286480

[38] Cahn, B. R. & Polich, J. Meditation states and traits: EEG, ERP, and neuroimaging studies. *Psychol. Bull.* **132**, 180–211 (2006). 16536641 10.1037/0033-2909.132.2.180

[39] Goyal, M. et al. Meditation programs for psychological stress and well-being: a systematic review and meta-analysis. *JAMA Intern. Med.* **174**, 357–368 (2014). 24395196 10.1001/jamainternmed.2013.13018 PMC4142584

[40] Frohlich, J., Simonian, N., Hanada, G., Kothe, C. & Reggente, N. Neural entrainment induced by periodic audiovisual stimulation: a large-sample EEG study. 10.1101/2023.10.25.563865 (2023).

[41] Vialatte, F.-B., Maurice, M., Dauwels, J. & Cichocki, A. Steady-state visually evoked potentials: Focus on essential paradigms and future perspectives. *Prog. Neurobiol.* **90**, 418–438 (2010). 19963032 10.1016/j.pneurobio.2009.11.005

[42] Johnson, M. A., Simonian, N. & Reggente, N. Lightening the mind with audiovisual stimulation as an accessible alternative to breath-focused meditation for mood and cognitive enhancement. *Sci. Rep.* **14**, 25553 (2024). 39462004 10.1038/s41598-024-75943-8 PMC11513117

[43] Reggente, N. VR for cognition and memory. in *Virtual Reality in Behavioral Neuroscience: New Insights and Methods*, Vol. 65 (eds Maymon, C., Grimshaw, G. & Wu, Y. C.) 189–232 (Springer International Publishing, Cham, 2023).

[44] Simonian, N. et al. Contrasting cognitive, behavioral, and physiological responses to breathwork vs. naturalistic stimuli in reflective chamber and VR headset environments. *PLoS Ment. Health* **2**, e0000269 (2025). 41661785 10.1371/journal.pmen.0000269 PMC12798627

[45] Cohen, S., Kamarck, T. & Mermelstein, R. A global measure of perceived stress. *J. Health Soc. Behav.* **24**, 385–396 (1983). 6668417

[46] Crumbaugh, J. C. & Maholick, L. T. An experimental study in existentialism: the psychometric approach to Frankl’s concept of noogenic neurosis. *J. Clin. Psychol.* **20**, 200–207 (1964). 14138376 10.1002/1097-4679(196404)20:2<200::aid-jclp2270200203>3.0.co;2-u

[47] Sutin, A. R., Luchetti, M., Stephan, Y., Sesker, A. A. & Terracciano, A. Purpose in life and stress: an individual-participant meta-analysis of 16 samples. *J. Affect. Disord.* **345**, 378–385 (2024). 38706462 10.1016/j.jad.2023.10.149 PMC11068359

[48] Boreham, I. D. & Schutte, N. S. The relationship between purpose in life and depression and anxiety: a meta-analysis. *J. Clin. Psychol.* **79**, 2736–2767 (2023). 37572371 10.1002/jclp.23576

[49] Krok, D. Can meaning buffer work pressure? An exploratory study on styles of meaning in life and burnout in firefighters. *Arch. Psychiatry Psychother.* **18**, 31–42 (2016).

[50] Hooker, S. A., Post, R. E. & Sherman, M. D. Awareness of meaning in life is protective against burnout among family physicians: a CERA study. *Fam. Med.* **52**, 11–16 (2020). 31689355 10.22454/FamMed.2019.562297

[51] Schaefer, S. M. et al. Purpose in life predicts better emotional recovery from negative stimuli. *PLoS ONE* **8**, e80329 (2013). 24236176 10.1371/journal.pone.0080329 PMC3827458

[52] Sarıcam H. Mediating role of self efficacy on the relationship between subjective vitality and school burnout in Turkish adolescents. *International Journal of Educational Researchers* **6**, 1–12 (2015).

[53] Chernenko, M. Burnout. in *The Rational Software Engineer* 63–75 (Apress, Berkeley, CA, 2023). 10.1007/978-1-4842-9795-7_5.

[54] Cavanagh, K. et al. A Randomised controlled trial of a brief online mindfulness-based intervention in a non-clinical population: replication and extension. *Mindfulness* **9**, 1191–1205 (2018). 30100934 10.1007/s12671-017-0856-1 PMC6061247

[55] Goddard, S. G., Stevens, C. J., Jackman, P. C. & Swann, C. A systematic review of flow interventions in sport and exercise. *Int. Rev. Sport Exerc. Psychol.* **16**, 657–692 (2021).

[56] Norcia, A. M., Appelbaum, L. G., Ales, J. M., Cottereau, B. R. & Rossion, B. The steady-state visual evoked potential in vision research: a review. *J. Vis.* **15**, 4 (2015). 26024451 10.1167/15.6.4 PMC4581566

[57] Quesnel, D. & Riecke, B. E. Are you awed yet? How virtual reality gives us awe and goose bumps. *Front. Psychol.* **9**, 2158 (2018). 30473673 10.3389/fpsyg.2018.02158 PMC6237834

[58] Sawada, K., Koike, H., Murayama, A., Nishida, H. & Nomura, M. Appreciation processing evoking feelings of being moved and inspiration: awe and meaning-making. *J. Creat.* **34**, 100076 (2024).

[59] Bai, Y. et al. Awe, daily stress, and elevated life satisfaction. *J. Pers. Soc. Psychol.* **120**, 837–860 (2021). 33764120 10.1037/pspa0000267

[60] Monroy, M. et al. Awe reduces depressive symptoms and improves well-being in a randomized-controlled clinical trial. *Sci. Rep.* **15**, 16453 (2025). 40355653 10.1038/s41598-025-96555-w PMC12069556

[61] Hendricks, P. S. Awe: a putative mechanism underlying the effects of classic psychedelic-assisted psychotherapy. *Int. Rev. Psychiatry* **30**, 331–342 (2018). 30260256 10.1080/09540261.2018.1474185

[62] Thaut, M. H. Neural basis of rhythmic timing networks in the human brain. *Ann. N. Y. Acad. Sci.* **999**, 364–373 (2003). 14681157 10.1196/annals.1284.044

[63] Brandmeyer, T. & Delorme, A. Meditation and neurofeedback. *Front. Psychol.* **4**, 688 (2013). 24109463 10.3389/fpsyg.2013.00688 PMC3791377

[64] Reggente, N. et al. Decoding depth of meditation: electroencephalography insights from expert Vipassana practitioners. *Biol. Psychiatry Glob. Open Sci.* **5**, 100402 (2025). 39660274 10.1016/j.bpsgos.2024.100402 PMC11629179

[65] Cohen, J. *Statistical Power Analysis for the Behavioral Sciences* (L. Erlbaum Associates, Hillsdale, N.J, 1988).

[66] Bakeman, R. Recommended effect size statistics for repeated measures designs. *Behav. Res. Methods* **37**, 379–384 (2005). 16405133 10.3758/bf03192707

[67] Preacher, K. J. & Hayes, A. F. Asymptotic and resampling strategies for assessing and comparing indirect effects in multiple mediator models. *Behav. Res. Methods* **40**, 879–891 (2008). 18697684 10.3758/brm.40.3.879

[68] Benjamini, Y. & Hochberg, Y. Controlling the false discovery rate: a practical and powerful approach to multiple testing. *J. R. Stat. Soc. Ser. B Stat. Methodol.* **57**, 289–300 (1995).

[69] Wang, R. & Ware, J. H. Detecting moderator effects using subgroup analyses. *Prev. Sci.* **14**, 111–120 (2013). 21562742 10.1007/s11121-011-0221-x PMC3193873

[70] JASP Team. *JASP (Version 0.95.4)[Computer Software]* (2025).

[71] Seabold, S. & Perktold, J. Statsmodels: econometric and statistical modeling with Python. *scipy* **7***,* 92-96 (2010).

[72] Hunter, J. D. Matplotlib: a 2D graphics environment. *Comput. Sci. Eng.* **9**, 90–95 (2007).

[73] Waskom, M. seaborn: statistical data visualization. *J. Open Source Softw.* **6**, 3021 (2021).

[74] Kristensen, T. S., Borritz, M., Villadsen, E. & Christensen, K. B. The Copenhagen Burnout Inventory: A new tool for the assessment of burnout. *Work Stress* **19**, 192–207 (2005).

[75] Demerouti, E. *Oldenburg Burnout Inventory*. 10.1037/t01688-000 (2012).

[76] Demerouti, E., Bakker, A. B., Nachreiner, F. & Schaufeli, W. B. The job demands-resources model of burnout. *J. Appl. Psychol.* **86**, 499–512 (2001). 11419809

[77] Maslach, C., Jackson, S. E. & Leiter, M. P. Maslach Burnout Inventory: Third edition. in *Evaluating Stress: A Book of Resources*. 191–218 (Scarecrow Education, Lanham, MD, US, 1997).

[78] Salmela-Aro, K., Rantanen, J., Hyvönen, K., Tilleman, K. & Feldt, T. Bergen Burnout Inventory: reliability and validity among Finnish and Estonian managers. *Int. Arch. Occup. Environ. Health* **84**, 635–645 (2011). 21082191 10.1007/s00420-010-0594-3

[79] Thørrisen, M. M. & Sadeghi, T. The Ten-Item Personality Inventory (TIPI): a scoping review of versions, translations and psychometric properties. *Front. Psychol.* **14**, 1202953 (2023). 37434881 10.3389/fpsyg.2023.1202953 PMC10330951

[80] Snyder, C. R. et al. The will and the ways: development and validation of an individual-differences measure of hope. *J. Pers. Soc. Psychol.* **60**, 570–585 (1991). 2037968 10.1037//0022-3514.60.4.570

[81] Ventura, M., Salanova, M. & Llorens, S. Professional self-efficacy as a predictor of burnout and engagement: the role of challenge and hindrance demands. *J. Psychol.* **149**, 277–302 (2015). 25590343 10.1080/00223980.2013.876380

[82] Wallston, K. A., Wallston, B. S. & DeVellis, R. Development of the Multidimensional Health Locus of Control (MHLC) scales. *Health Educ. Monogr.* **6**, 160–170 (1978). 689890 10.1177/109019817800600107

[83] Connor, K. M. & Davidson, J. R. T. Development of a new resilience scale: the Connor-Davidson Resilience Scale (CD-RISC). *Depress. Anxiety* **18**, 76–82 (2003). 12964174 10.1002/da.10113

[84] Bartone, P. T., Ursano, R. J., Wright, K. M. & Ingraham, L. H. The impact of a military air disaster on the health of assistance workers. A prospective study. *J. Nerv. Ment. Dis.* **177**, 317–328 (1989). 2723619 10.1097/00005053-198906000-00001

[85] Heuchert, J. P. & McNair, D. M. *Profile of Mood States 2nd Edition*^*T*^^*M*^. 10.1037/t05057-000 (2012).

[86] Setty, J. V., Srinivasan, I., Radhakrishna, S., Melwani, A. M. & DR, M. K. Use of an animated emoji scale as a novel tool for anxiety assessment in children. *J. Dent. Anesth. Pain Med.* **19**, 227–233 (2019). 31501781 10.17245/jdapm.2019.19.4.227 PMC6726885

[87] Watson, D., Clark, L. A. & Tellegen, A. Development and validation of brief measures of positive and negative affect: the PANAS scales. *J. Pers. Soc. Psychol.* **54**, 1063–1070 (1988). 3397865 10.1037//0022-3514.54.6.1063

[88] Engeser, S. & Rheinberg, F. Flow, performance and moderators of challenge-skill balance. *Motiv. Emot.* **32**, 158–172 (2008).

[89] Spielberger, C. D. *State-Trait Anxiety Inventory for Adults*. 10.1037/t06496-000 (2012).

[90] Roseman, L. et al. Emotional breakthrough and psychedelics: Validation of the Emotional Breakthrough Inventory. *J. Psychopharmacol.* **33**, 1076–1087 (2019). 31294673 10.1177/0269881119855974

[91] Lau, M. A. et al. The Toronto Mindfulness Scale: development and validation. *J. Clin. Psychol.* **62**, 1445–1467 (2006). 17019673 10.1002/jclp.20326

