# Supplementary material for: Alpha and theta audiovisual interventions in a reflective chamber demonstrate acute effects on stress and burnout
Source: NPJ Digit Med. 2026 Mar 28;9:390. doi: 10.1038/s41746-026-02555-z (PMC13194715; doi:10.1038/s41746-026-02555-z)
Supplement: Supplementary file 1 — Supplementary Materials [file 41746_2026_2555_MOESM1_ESM.docx]

**Supplementary Materials**

**Results**

**Baseline Mood Disturbance as a Predictor of Intervention Effects**

| **POMS TMD Group** | **Measure** | **t-statistic** | **Effect size (Cohen’s *d)*** | **Bonferroni Corrected p-value** |
| --- | --- | --- | --- | --- |
| High | PIL Delta | *t(65)* = -2.343 | -0.815 | 0.023 |
|  | FSS Total Delta | *t(65) =* -2.358 | 0.824 | 0.021 |
|  | FSS Fluency of Performance Delta | *t(65) =* -1.153 | -0.403 | 0.253 |
|  | STAI Delta | *t(65) = 0.835* | 0.292 | 0.407 |
|  | PSS Delta | *t(65) =* -1.621 | -0.567 | 0.110 |
|  | PANAS Negative Affect Delta | *t(65) =* 0.463 | 0.162 | 0.645 |
|  | PANAS Positive Affect Delta | *t(65) =* 0.324 | 0.113 | 0.747 |
|  | POMS Depression Delta | *t(65) =* 1.011 | 0.353 | 0.316 |
|  | POMS Tension Delta | *t(65) =* 1.032 | 0.361 | 0.306 |
|  | POMS Anger Delta | *t(65) =* 0.430 | 0.150 | 0.668 |
|  | POMS Confusion Delta | *t(65) =* 0.750 | 0.262 | 0.456 |
|  | SVS Delta | *t(65) =* 0.333 | 0.117 | 0.740 |

**Supplementary Table 1. POMS TMD High Group Analyses.** Independent samples t-tests comparing intervention effects (change scores) between alpha and theta conditions among participants with high baseline POMS Total Mood Disturbance (above median split). Negative Cohen's d values indicate greater improvement in the theta condition; positive values indicate greater improvement in the alpha condition. Bonferroni correction applied for multiple comparisons. Significant differences (p < .05) are observed for Purpose in Life and Total Flow Score, favoring theta-frequency stimulation.

| **POMS TMD Group** | **Measure** | **t-statistic** | **Effect size (Cohen’s *d)*** | **Bonferroni Corrected p-value** |
| --- | --- | --- | --- | --- |
| Low | PIL Delta | *t(65)* = 0.486 | 0.162 | 0.629 |
|  | FSS Total Delta | *t(65) =* -2.358 | 0.824 | 0.021 |
|  | FSS Fluency of Performance Delta | *t(65) =* -1.153 | -0.403 | 0.253 |
|  | STAI Delta | *t(65) =* -0.658 | -0.219 | 0.513 |
|  | PSS Delta | *t(65) =* -1.215 | -0.405 | 0.229 |
|  | PANAS Negative Affect Delta | *t(65) =* -0.235 | -0.078 | 0.815 |
|  | PANAS Positive Affect Delta | *t(65) =* -0.500 | -0.167 | 0.618 |
|  | POMS Depression Delta | *t(65) =* 0.902 | 0.301 | 0.370 |
|  | POMS Tension Delta | *t(65) =* -0.518 | -0.173 | 0.606 |
|  | POMS Anger Delta | *t(65) =* 0.495 | 0.165 | 0.622 |
|  | POMS Confusion Delta | *t(65) =* 0.062 | 0.021 | 0.950 |
|  | SVS Delta | *t(65) =* -0.373 | -0.124 | 0.711 |

**Supplementary Table 2. POMS TMD Low Group Analyses.** Independent samples t-tests comparing intervention effects (change scores) between alpha and theta conditions among participants with low baseline POMS Total Mood Disturbance (below median split). Negative Cohen's d values indicate greater improvement in the theta condition; positive values indicate greater improvement in the alpha condition. Bonferroni correction applied for multiple comparisons. No significant differences emerged between conditions for participants with low baseline distress.

| **Specific Measure** | **F-statistic (1, 29)** | **p-value** | **Effect Size**  **(***η²_p_; η²_G_;* Cohen’s *d*^^[[1]](#footnote-1)^^**)** | **Bonferroni Corrected p-value** |
| --- | --- | --- | --- | --- |
| HR Time Effect | 0.067 | 0.798 | 0.002; 1.21x10^-4^ | N/A |
| HR Condition Effect | 0.284 | 0.598 | 0.01; 0.009 | N/A |
| HR Time × Condition | 0.5184 | 0.447 | 0.018; 8.661x10^-4^ | N/A |
| HRV Time Effect | 0.257 | 0.616 | 0.009; 0.001 | N/A |
| HRV Condition Effect | 3.14 | 0.087 | 0.098; 0.083 | N/A |
| HRV Time × Condition | 0.117 | 0.735 | 0.004; 6.423x10^-4^ | N/A |

**Supplementary Table 3. Physiology changes pre- to post-intervention**

For HR, no significant main effect of time (F(1, 29) = 0.067, *p* = 0.798, *η^2^_p_* = 0.002) or significant interaction between time and group (F(1, 29) = 0.5184, p = 0.447, *η^2^_p_* = 0.018) were observed. Additionally, the two groups were not significantly different overall (F(1, 29) = 0.284, p = 0.598, *η^2^_p_* = 0.01). For HRV, no significant main effect of time (F(1, 29) = 0.257, p = 0.616, *η^2^_p_* = 0.009) or significant interaction between time and group (F(1, 29) = 0.117, p = 0.735, *η^2^_p_* = 0.004) were observed. Additionally, the two groups were not significantly different overall (F(1, 29) = 3.14, p = 0.087, *η^2^_p_* = 0.098). The two groups showed no statistical difference in post-rest HR (*t(29)* = -0.665, *p* = 0.511, *d* = -0.24) or post-rest HRV (*t(29)* = 1.81, *p* = 0.08, *d* = 0.655.

| **Measure** | **F-statistic (1, 64)** | **p-value** | **Effect Size**  **(***η²_G_;* Cohen’s *d****)** | **Bonferroni Corrected p-value** |
| --- | --- | --- | --- | --- |
| Alpha Time Effect | 0.102 | 0.750 | 3.915x10^-5^ | N/A |
| Alpha Condition Effect | 4.151 | 0.046 | 0.060; 0.496 [95% CI, 0.002 – 0.989] | 0.82 |
| Alpha Time x Condition Effect | 0.156 | 0.69 | 5.9 x 10^-5^ | N/A |
| Theta Time Effect | 0.451 | 0.505 | 2.8 x 10^-4^ | N/A |
| Theta Condition Effect | 1.95 | 0.167 | 0.028 | N/A |
| Theta Time x Condition Effect | 0.164 | 0.687 | 1.02 x 10^-4^ | N/A |

**Supplementary Table 4. EEG power band changes pre- to post-intervention.**

Analysis of EEG band powers revealed marginally significant differences in post-rest alpha power (*t(64)* = 2.08, *p* = 0.044, *d* = 0.50 [95% CI, 0.0091 – 0.9895]), however, this did not survive Bonferroni correction (*p_bonf_* = 0.26). There was also no significant difference in post-rest theta power between groups found (*t(64)* = 1.42, *p* = 0.169, *d* = 0.34 [95% CI, -0.144 – 0.829]).

| **Measure** | **t-statistic** | **p-value** | **Effect size (Cohen’s *d)*** | **Bonferroni Corrected p-value** |
| --- | --- | --- | --- | --- |
| Heart Rate (HR) | *t(29)* = -0.665 | 0.511 | -0.24 | 0.57 |
| Heart Rate Variability (HRV) | *t(29)* = 1.81 | 0.08 | 0.66 | 0.20 |
| Alpha Power | *t(64)* = 2.08 | *p* = 0.044 | 0.50 [95% CI, 0.0091 – 0.9895] | 0.26 |
| Theta Power | *t(64)* = 1.42 | *p* = 0.169 | 0.34 [95% CI, -0.144 – 0.829] | 0.97 |
| Alpha/Beta Power | *t(64)* = 2.12 | *p* = 0.041 | 0.51 [95% CI, 0.018 – 0.999] | 0.24 |
| Theta/Beta Power | *t(64)* =0.26 | *p* = 0.79 | 0.064 [95% CI, -0.419 – 0.5467] | 1.0 |
| Gamma/Alpha Power | *t(64)* = -2.51 | *p* = 0.017 | -0.6 [95% CI, -1.094 – -0.1069 | 0.10 |
| (Alpha+Theta)/(Beta/Gamma) | *t(64)* = -0.025 | *p* = 0.98 | -0.006 [95% CI, -0.489 – 0.476] | 1.0 |

**Supplementary Table 5. Results physiological and EEG group effect analyses.**

Looking at EEG band power ratios, significant differences in post-rest alpha power, alpha/beta power, and gamma/alpha power were tentatively found between the two groups. The exploratory analysis revealed a significant difference in post-rest alpha power (*t(64)* = 2.08, *p* = 0.044, *d* = 0.50 [95% CI, 0.009 – 0.989]) and alpha/beta power (*t(64)* = 2.12, *p* = 0.041, *d* = 0.51 [95% CI, 0.018 – 0.999]). The two groups also tentatively showed a significant difference in gamma/alpha power (*t(64)* = -2.51, *p* = 0.017, *d* = -0.6 [95% CI, -1.094 – -0.1069]). However, after correcting for multiple comparisons, these differences did not survive Bonferroni correction (*p_Bonf_* = 0.26, 0.24, and 0.10, respectively). No significant differences were observed in theta power (*t(64)* = 1.42, *p* = 0.169, *d* = 0.34 [95% CI, -0.144 – 0.829]), theta/beta power (*t(64)* = 0.26, *p* = 0.79, *d* = 0.064 [95% CI, -0.419 – 0.5467]), or (alpha+theta)/(beta+gamma) power (*t(64)* = -0.025, *p* = 0.98, *d* = -0.006 [95% CI, -0.489 – 0.476]).

| **Measure** | **Time Effect** | **Group Effect** | **Time x Group Interaction** | **Key Finding** |
| --- | --- | --- | --- | --- |
| Alpha/Beta Power | F(1, 64) = 0.158, *p* = 0.69,  *η^2^_p_* = 0.0025,  *η²_G_* = 0.0 | *F(1, 64)* = 3.94,  *p* = 0.052,  *p_FDR_* = 0.982  *η^2^_p_* = 0.058,  *η²_G_* = 0.053 | F(1, 64) = 0.553,  *p* = 0.459,  *η^2^_p_* = 0.0086,  *η²_G_* = 0.0001 | No significant differences |
| Gamma/Alpha Power | F(1, 64) = 0.2335,  *p* = 0.631,  *η^2^_p_* = 0.0036,  *η²_G_* = 0.0001 | *F(1, 64)* = 4.99,  *p_FDR_* = 0.052,  *η^2^_p_* = 0.072,  *η²_G_* = 0.068 | F(1, 64) = 0.81,  *p* = 0.3707,  *η^2^_p_* = 0.0125,  *η²_G_* = 0.0002 | No significant differences |
| (Alpha+Theta)/(Beta+Gamma) | F(1, 64) = 1.44,  *p* = 0.235,  *η^2^_p_* = 0.022,  *η²_G_* = 0.0003 | F(1, 64) = 0.0007,  *p* = 0.973,  *η^2^_p_* = 0.0,  *η²_G_* = 0.0 | F(1 ,64) = 0.0503,  *p* = 0.823,  *η^2^_p_* = 0.0008,  *η²_G_* = 0.0 | No significant differences |
| Theta/Beta Power | F(1, 64) = 0.0016,  *p* = 0.968,  *η^2^_p_* = 0.0,  *η²_G_* = 0.0 | F(1, 64) = 0.0071,  *p* = 0.933,  *η^2^_p_* = 0.0001,  *η²_G_* = 0.0 | F(1, 64) = 0.808,  *p* = 0.372,  *η^2^_p_* = 0.0125,  *η²_G_* = 0.0002 | No significant differences |

**Supplementary Table 6. EEG band power ratio changes from pre- to post-intervention.**

**EEG Pre vs. Post**

Repeated measures ANOVA were also utilized for the EEG band power and band power ratio analyses pre- and post-intervention. Alpha power had a significant difference between groups pre-post measurements (*F(1, 64)* = 4.15, *p* = 0.046, *η^2^_p_* = 0.061). No significant main effect of time (*F(1, 64)* = 0.102, p = 0.750, *η^2^_p_* = 0.002) or interaction between time and group (*F(1, 64)* = 0.156, p = 0.694, *η^2^_p_* = 0.002) were found. No significant main effect of time or interaction with groups were observed for theta power.

For the power band ratios, the exploratory analysis revealed a marginally significant difference between the Alpha and Theta groups for alpha/beta power looking at pre-rest and post-rest together (*F(1, 64)* = 3.94, p = 0.052, *η^2^_p_* = 0.058). No significant main effect of time or interaction with groups were observed for alpha/beta power. Similarly, for gamma/alpha power, the two groups were tentatively significantly different overall (*F(1, 64)* = 4.99, p = 0.029, *η^2^_p_* = 0.072). It is important to note this difference did not survive Bonferroni correction for multiple comparisons. However, no significant main effect of time or interaction with groups were observed for gamma/alpha power. Looking at (alpha+theta)/(beta+gamma) power band ratio, there were no significant main effect of time (*F(1, 64)* = 1.4398, *p* = 0.235, *η^2^_p_* = 0.022) or interaction between time and group (*F(1, 64)* = 0.0503, *p* = 0.823, *η^2^_p_* = 0.001). Additionally, the two groups were not significantly different overall (*F(1, 64)* = 6.8x10-4, *p* = 0.979, *η^2^_p_* = 0.000). For anxiolytic effects in the theta/beta power band, there were no significant main effect of time (*F(1, 64)* = 0.0434, *p* = 0.836, *η^2^_p_* = 0.001) or interaction between time and group (*F(1, 64)* = 0.5265, *p* = 0.471, *η^2^_p_* = 0.008). Additionally, the two groups were not significantly different overall (*F(1, 64)* = 0.982, *p* = 0.326, *η^2^_p_* = 0.015).

**
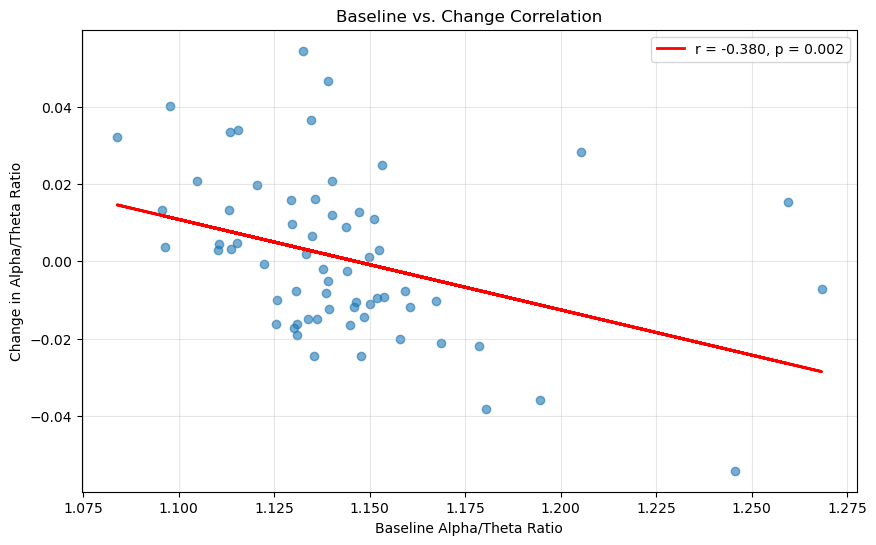
**

**Supplementary Figure 1. Baseline Alpha/Theta pre-rest vs. Change Correlation.** Scatterplot shows a significant negative correlation (*r* = -0.380, *p* = 0.002) between pre-intervention alpha/theta ratios and change scores. Participants with higher baseline ratios experienced greater decreases, while those with lower baseline ratios showed greater increases, suggesting a regression-to-the-mean effect where the intervention normalizes alpha/theta ratios rather than producing uniform directional changes.

No significant EEG × condition interaction effects survived correction for multiple comparisons (all *p_FDR_* > 0.05). At the uncorrected level, several interaction patterns emerged that suggested potential moderation of treatment outcomes by baseline neurophysiological state. For example, alpha/theta ratio predicted POMS Anger delta scores more strongly in the theta condition (β = -78.99, p = 0.019, R² = 17.6%) than in the alpha condition (β = -8.16, p = 0.591, R² = 0.9%), indicating that participants with higher baseline alpha/theta ratios showed greater anger reduction in the theta group. Similarly, exploratory moderation effects were observed for POMS Confusion (β = -55.689, p = 0.037, R² = 9.2%), POMS Total Mood Disturbance (β = 13.522, p = 0.039, R² = 6.9%), and POMS Vigor (β = -3.682, p = 0.045, R² = 7.2%).

However, after applying False Discovery Rate (FDR) correction, none of these effects retained statistical significance (all *p_FDR_* > 0.42). Thus, these findings should be regarded as exploratory patterns rather than confirmatory evidence. While the effect sizes (R² = 6–18%) and consistency of directionality suggest possible mechanisms by which baseline alpha/theta ratios could moderate treatment response, firm conclusions cannot be drawn. Future studies with larger samples and greater power to withstand correction for multiple comparisons are needed to determine whether these preliminary patterns reflect genuine neurophysiological moderators of treatment outcomes.

To determine whether intervention effects on alpha/theta ratios reflected uniform directional changes or instead depended on participants’ initial neurophysiological state, we examined the relationship between baseline alpha/theta ratios and subsequent changes across the intervention. This analysis provides insight into whether the intervention acts as a general enhancer/suppressor of oscillatory activity or functions as a normalizing mechanism, shifting extreme values toward the group mean.

Baseline-Change Relationship correlation analysis examined the relationship between baseline alpha/theta ratios and intervention-related changes in alpha/theta ratios. The results, shown in Figure 6, revealed a significant negative correlation (*r* = -.38, *p* = 0.002, 95% CI [-.572, -.148]), indicating that participants with higher baseline alpha/theta ratios demonstrated greater decreases following the intervention, while those with lower baseline ratios showed greater increases.

This pattern suggests a regression-to-the-mean effect, where the intervention may normalize alpha/theta ratios rather than uniformly increasing them across all participants. Alpha/Theta Change and Anxiety Outcomes To examine whether changes in alpha/theta ratios were directly associated with therapeutic benefits, we analyzed the correlation between alpha/theta change scores and STAI State anxiety changes. Results showed no significant relationship (*r* = .087, *p* = .495, 95% CI [-.162, .326]), suggesting that the magnitude of alpha/theta change was not predictive of anxiety reduction. These findings indicate that baseline neurophysiological state may be more important than intervention-induced changes in predicting treatment outcomes. The lack of correlation between alpha/theta changes and anxiety improvements, combined with the regression-to-the-mean pattern, suggests that the intervention's therapeutic mechanism may involve normalizing brain states rather than producing uniform directional changes in alpha/theta ratios.

1. [↑](#footnote-ref-1)
